# Impact on Clinical and Cost Outcomes of a Centralized Approach to Acute Stroke Care in London: A Comparative Effectiveness Before and After Model

**DOI:** 10.1371/journal.pone.0070420

**Published:** 2013-08-01

**Authors:** Rachael Maree Hunter, Charles Davie, Anthony Rudd, Alan Thompson, Hilary Walker, Neil Thomson, James Mountford, Lee Schwamm, John Deanfield, Kerry Thompson, Bikash Dewan, Minesh Mistry, Sadik Quoraishi, Stephen Morris

**Affiliations:** 1 Research Department of Primary Care and Population Health, University College London, London, United Kingdom; 2 Department of Clinical Neurosciences, Royal Free London NHS Foundation Trust, London, United Kingdom; 3 Division of Health and Social Care Research, King’s College London, London, United Kingdom; 4 National Institute for Health Research Comprehensive Biomedical Research Centre, Guy’s and St Thomas’ NHS Foundation Trust, London, United Kingdom; 5 University College London Institute of Neurology, University College London, London, United Kingdom; 6 North Central London CardioVascular & Stroke Network, London, United Kingdom; 7 London Ambulance Service NHS Trust, London, United Kingdom; 8 UCLPartners, London, United Kingdom; 9 Department of Neurology, Massachusetts General Hospital, Boston, Massachusetts, United States of America; 10 Centre for Cardiovascular Prevention and Outcomes, University College London, London, United Kingdom; 11 UCL Medical School, University College London, London, United Kingdom; 12 Department of Applied Health Research, University College London, London, United Kingdom; University of Toronto, Canada

## Abstract

**Background:**

In July 2010 a new multiple hub-and-spoke model for acute stroke care was implemented across the whole of London, UK, with continuous specialist care during the first 72 hours provided at 8 hyper-acute stroke units (HASUs) compared to the previous model of 30 local hospitals receiving acute stroke patients. We investigated differences in clinical outcomes and costs between the new and old models.

**Methods:**

We compared outcomes and costs ‘before’ (July 2007–July 2008) vs. ‘after’ (July 2010–June 2011) the introduction of the new model, adjusted for patient characteristics and national time trends in mortality and length of stay. We constructed 90-day and 10-year decision analytic models using data from population based stroke registers, audits and published sources. Mortality and length of stay were modelled using survival analysis.

**Findings:**

In a pooled sample of 307 patients ‘before’ and 3156 patients ‘after’, survival improved in the ‘after’ period (age adjusted hazard ratio 0.54; 95% CI 0.41–0.72). The predicted survival rates at 90 days in the deterministic model adjusted for national trends were 87.2% ‘before’ % (95% CI 86.7%–87.7%) and 88.7% ‘after’ (95% CI 88.6%–88.8%); a relative reduction in deaths of 12% (95% CI 8%–16%). Based on a cohort of 6,438 stroke patients, the model produces a total cost saving of £5.2 million per year at 90 days (95% CI £4.9-£5.5 million; £811 per patient).

**Conclusion:**

A centralized model for acute stroke care across an entire metropolitan city appears to have reduced mortality for a reduced cost per patient, predominately as a result of reduced hospital length of stay.

## Introduction

Stroke is a leading cause of mortality and disability worldwide. [1] Organized inpatient stroke care has been shown to decrease morbidity and mortality. In a review of 31 randomized controlled trials comparing stroke unit (SU) care with an alternative service, SUs were associated with lower risk of death at one year (odds ratio (OR) 0.86; P = 0.02), of death or institutionalized care (OR 0.82; P = 0.0006) and death or dependency (OR 0.82; P = 0.001) [2]. A large observational study in New York State showed improved mortality at one day, 30 days (2.5% absolute reduction in adjusted 30 day all-cause mortality) and one year in patients treated in designated stroke centers compared to patients admitted to non-designated hospitals after acute ischemic stroke [3]. While there is evidence that outcomes are better in more organized services [2], little is known about the benefits of intensive specialist stroke care in the first 72 hours after stroke. It is also not known whether centralizing acute stroke care to a small number of high volume specialist centers can provide better outcomes across a large metropolitan city and whether such a model is cost effective.

In 2006 a report by Professor Lord Ara Darzi recommended greater specialized acute care delivered in dedicated, high-volume stroke units [4]. The aim was to provide a uniform high-quality standard of care, including rapid assessment and treatment for all stroke patients in London irrespective of location, 24 hours a day, seven days a week. At this time, the dominant model of care for acute stroke patients in London as well as the rest of England centered on local hospital care. Thirty of London’s hospital had designated stroke units (SUs), but a national audit of all stroke units in England in 2008 identified that improvements in stroke care in London had been slower than the rest of England [5].

Following Lord Darzi’s recommendation, a London-specific stroke strategy was published in 2008 [6]. This made a number of recommendations regarding the prevention of stroke, provision of acute stroke services, and rehabilitation of stroke patients. One of the most significant recommendations was the rapid implementation of a new model of acute care with a small number of highly specialist units providing hyper-acute stroke care in the first 72 hours for all suspected stroke patients and, in addition, a larger number of acute stroke units with enhanced specialist care and multi-therapy rehabilitation for those patients requiring ongoing in-patient care beyond 72 hours.

The stroke care model was co-created through a series of events with key stakeholders, clinical experts, patients and carers as well as representatives from carer groups.

For a unit to be accredited as a hyper-acute stroke unit (HASU) or SU it had to meet pre-defined service specifications, including high minimum staffing levels, assessed by an independent expert panel. The model was supported by extra investment, via an enhanced tariff for each patient [7].

**Figure 1:**
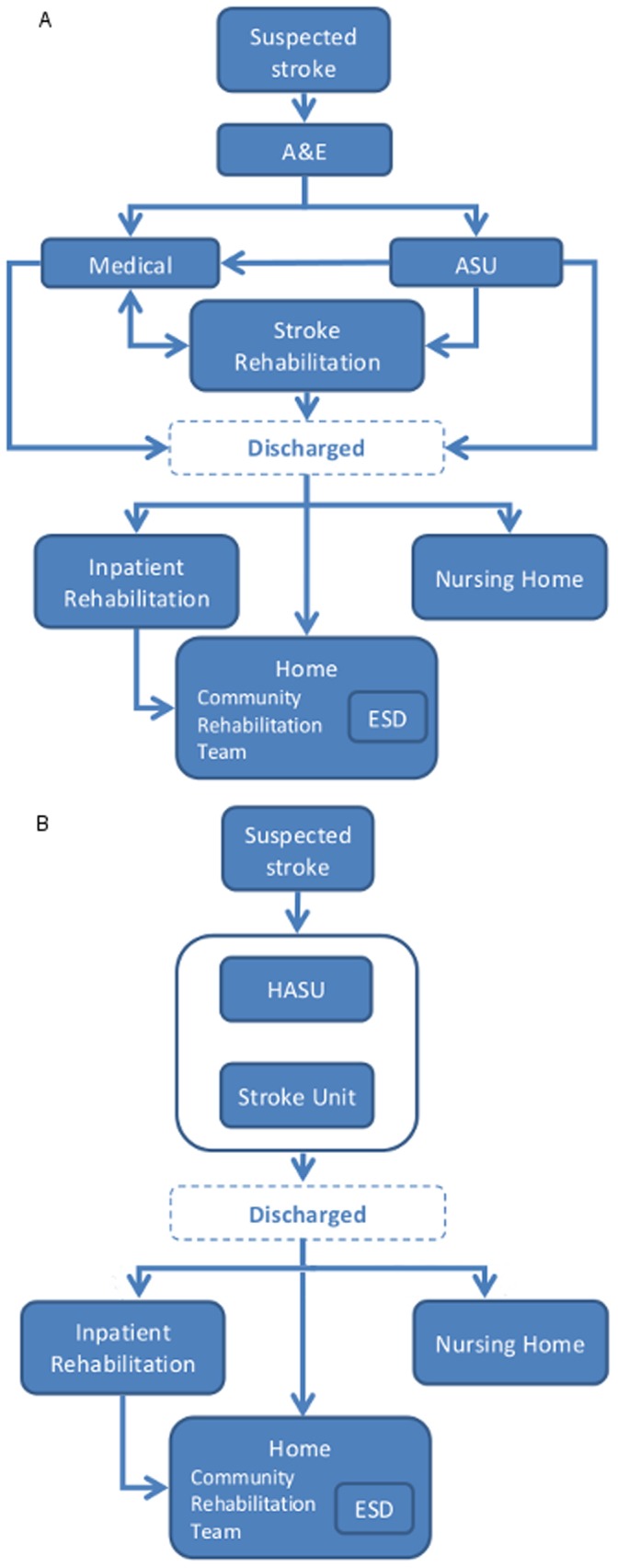
Stylized depiction of new and previous stroke model. A Previous model (‘before’). B New model (‘after’). Abbreviations: A&E – Accident and Emergency Department; ASU – Acute Stroke Unit; HASU – Hyper Acute Stroke Unit; ESD – Early Supported Discharge.

The new model for stroke care was introduced in July 2010. Eight HASUs were created to provide faster response times to a suspected stroke, and continual access to specialist care throughout the first 72 hours. This was complemented by SUs for on-going inpatient care if necessary after 72 hours (see Figure 1 for a stylized depiction of the ‘before’ and ‘after’ stroke models).

We sought to determine the clinical outcomes (adjusted all-cause mortality) and cost-effectiveness of the new London model at 90 days and 10 years after admission to hospital using two cost-effectiveness models. Randomised trials in this area are challenging if not impossible because of the nature of the intervention, which is a city-wide reconfiguration of services. Hence, we used a before-and-after study design, adjusting for national trends and comparing costs and outcomes of stroke patients in London between July 2007–June 2008 and July 2010–June 2011 using a population based stroke register and audit data. The ‘before’ period was the most recent period before the new model was introduced in any part of London.

## Methods

### Ethics

Patients or their relatives provide informed consent for collection and storage of their data for the South London Stroke Register (SLSR). All other data sources contained de-identified patient data collected for the purpose of service evaluation or audit and hence ethics approval was not required.

### Models of Acute Stroke Service Delivery

A stylized depiction of the stroke models before and after reconfiguration are in Figure 1 and Supplementary Appendix Figure S1 in File S1.

### Study Population

Our study population was patients who had an ischemic or hemorrhagic stroke in London between July 2007–June 2008 and July 2010–June 2011. The main data sources were: the SLSR; an audit from two North London hospitals; the London Minimum Dataset (LMDS); the Stroke Improvement National Audit Programme (SINAP); the national Sentinel Stroke Audit; and, the London Ambulance Service.

The SLSR is a population-based stroke prospective registry recording all first-ever strokes in patients of all ages living in an area of South London. [8], [9] Data on long-term outcomes and utilization of hospital and community care services up to July 2011 were extracted for patients with a stroke-related hospital admission date from July 2007–June 2008 for the ‘before’ period and July 2010–January 2011 for ‘after’. Stroke diagnosis was ascertained by a physician and patients were followed up via structured questionnaire at 48 hours and three months, and yearly thereafter. [9].

Outcomes and resource data for the inpatient component of the stroke pathway were collected from a retrospective audit of all patients admitted to two large North London hospitals with a diagnosis of stroke between April–June 2008 and April–June 2011.

Data for the ‘after’ period only were obtained from SINAP and the LMDS. These retrospective datasets included all patient admissions for stroke from January–June 2011 in London, and combined they covered the stroke pathway from hospital admission to discharge, with data on mortality and length of hospital stay. Because they were available for the ‘after’ period only, we ran models with and without these data.

Sentinel is the national stroke audit conducted from April to June every two years, covering the stroke pathway from stroke onset to discharge from acute care, based on data for the first 60 consecutive stroke cases in all acute hospitals in England. Data from the Sentinel audits in 2008 and 2010 were used to adjust for changes in mortality and LOS that occurred elsewhere in the country during this period. [5], [10].

Information on time from emergency call to arrival at hospital was provided by the London Ambulance Service (LAS) for 23,365 stroke patients ‘before’ (January 2005–March 2008), identified by their illness code, and 7,375 stroke patients ‘after’ (July 2010–May 2011), identified using a pre-hospital stroke screen.

See Table S1 in File S1 for more details.

### Outcome Measures

Survival time was measured from the first date of admission to hospital until date of death. The censoring point for patients with no death record was the date of hospital discharge (North London dataset) or the last update date (SLSR and SINAP/LMDS).

Date of admission, date of discharge and discharge location were available in all three datasets, and used to calculate LOS and discharge location by ward type.

‘Health States’ in the 90-day model were based on ward type and discharge location. Each state was assigned a mean cost per patient per day and utility score. We accounted for differences in costs and admission rates by type of stroke and thrombolysis rates.

Utilities to calculate quality-adjusted life-years (QALYs) were derived from the Barthel index, available in the SLSR and North London data, using a new UK algorithm [11].

We calculated mean ambulance travel times and resources required from time of emergency call to arrival at hospital.

For further details on utilities and unit costs see Table S2–S3 in File S1.

### Statistical Analysis

To investigate differences in ‘before’ and ‘after’ survival, we ran semi-parametric (Cox proportional hazards) and parametric (Weibull) survival models. We also controlled for age in these models. We only controlled for age because no other variables had a significant impact on the results, and we wanted a parsimonious model to more easily incorporate the results into the economic analysis. The time dependent hazard of death from hospital admission was calculated using separate survival models for each period to allow different shape parameters for the two mortality estimates.

Time-dependent movements between wards and discharge locations for the 90-day model were calculated similarly using parametric survival models, controlling for destination ward and discharge location.

### Measuring Cost-effectiveness

Cost-effectiveness was measured in terms of the incremental cost per death averted at 90 days after hospital admission, and the incremental cost per QALY gained at 90 days and 10 years after hospital admission. We constructed two time-dependent Markov models [12] (see Methods S1–S2 in File S1), each with a hypothetical population of 6,438 strokes in London, based on 2009/10 *Hospital Episode Statistics*. [13] The first model covers time from admission to 90 days (one day cycles) and the second from 90 days to 10 years (90 day cycles). Transition probabilities were time-dependent and varied by days from admission to hospital. Transition probabilities for the 90 day to 10 year model are reported in Table S4 in File S1 and corresponding Barthel Index categories are in Table S5 in File S1. See Figure S2 in File S1 for a stylized depiction of the 90 days to 10 years model.

Costs were measured using an English National Health Service and Personal Social Services perspective, [14] in 2010/11 UK£. We measured costs of transport, acute hospitalisation, imaging and surgical interventions, staff contacts, medications during acute hospitalisation and post-discharge care (see Table S1 in File S1).

To account for the before-and-after nature of our analysis we adjusted for national trends in mortality and LOS, the main drivers of costs and outcomes, using Sentinel Stroke Audit data. We reduced the number of deaths at 30 days in the ‘before’ period to reflect that across England, excluding London, there was a drop in the number of stroke patients that died at 30 days by 2.4 percentage points between 2008 and 2010 (20.7% vs. 18.3%). [10], [5] We reduced the LOS on the SU in the before period to reflect that across England, excluding London, there was a drop in mean LOS on a SU of 5.0 days between 2008 and 2010 (23.1 days vs. 18.1 days). [10], [5].

We undertook a probabilistic sensitivity analysis based on 10,000 simulations of the models. We used this to compute confidence intervals for point estimates of cost-effectiveness and to draw cost-effectiveness acceptability curves. In the 10 year model costs and QALYs were discounted at an annual rate of 3.5%. [14].

See Methods S3 and Table S6 in File S1 for sensitivity analysis details.

The reporting of this study conforms with the EVEREST statement.

## Findings

The sample for the 90-day model comprised 307 patients ‘before’, 3,156 patients ‘after’ (Table 1); we also present results for ‘after’ using 319 North London and SLSR patients only (the datasets available in the ‘before’ period). The three samples were similar in terms of age, gender and stroke type (Table 1).

**Table 1:** Comparison of ‘before’ and ‘after’ sample and resource use.

	Before Period	After Period (1)	After Period (2)
	North London and SLSR	North London, SLSR and SINAP/LMDS	North London and SLSR only
**Databases (n)**			
SLSR	205	100	100
North London Database	102	219	219
SINAP/LMDS		2,837	
Total	307	3,156	319
**Age (Mean (SD)**	71(15.2)	72.8(14.86)	71.6(15.2)
**Gender (% male (n))**	51%(156)	51%(1612)	53%(315)
**Stroke type (% (n))**			
Ischemic	85%(254)	88%(2,768)	86%(212)
Hemorrhagic	15%(44)	12%(371)	14%(35)
**Thrombolysis (intravenous)** **(% (n))**	5% (61) [10]	13% (412)	12%(31)
**LOS: first ward admitted (median days (n))**			
HASU		3 (2352)	2(207)
ASU	4 (141)		
SU		3 (425)	12(7)
Stroke rehabilitation	4 (45)		
Medical ward	2 (71)	3 (303)	2(42)
Surgical ward	5 (9)	3 (3)	3(3)
ICU	4·5 (16)	4 (47)	3·5(6)
**Imaging and surgical interventions (% (n))**			
Head CT Scan (non-contrast)*	95% (279)	94% (2935)	92%(195)
Head MRI Scan (non-contrast)*	51% (139)	68% (121)	68% (121)
CT Angiography*	40% (104)	63% (133)	63% (133)
Echocardiogram (transthoracic)*	28% (80)	49% (111)	49% (111)
Carotid Stenting	11% (11)	14% (20)	14% (20)
Neurosurgery	6% (6)	1% (3)	1% (3)
**Outcomes – Admission**			
Barthel Index (Mean (SD))	9·3 (7·6)	10·7 (7·8)	10·7 (7·8)
Health Utility [11] (Mean (SD))	0·23 (0·31)	0·30 (0·32)	0·30 (0·32)

* Patients may receive more than 1.

**Abbreviations: SLSR –** South London Stroke Register; **SINAP –** Stroke Improvement National Audit Programme; **LMDS –** London Minimum Dataset; **SD –** Standard Deviation**; LOS** **–** Length of Stay; **HASU –** Hyper Acute Stroke Unit; **ASU –** Acute Stroke Unit; **SU –** Stroke Unit; **ICU –** Intensive Care Unit; **CT –** Computerised Tomography; **MRI –** Magnetic Resonance Imaging.

**Figure 2:**
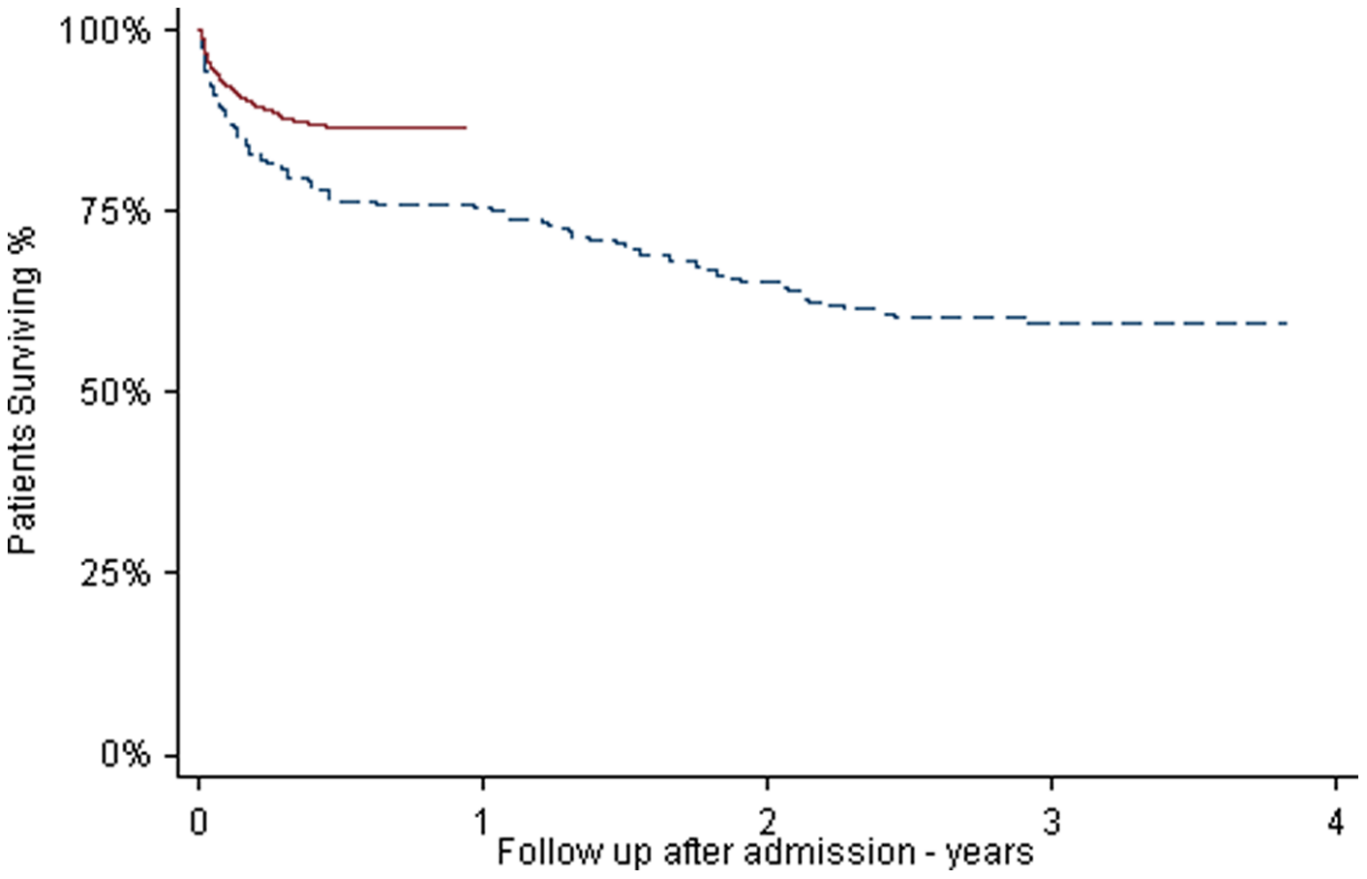
Kaplan-Meier estimates of time from admission to hospital to death comparing ‘before’ and ‘after’. **- - - - -** Before. **———** After.

The adjusted hazard ratio of dying from stroke ‘after’ versus ‘before’ was 0.54 (95% CI 0.41–0.72; Figure 2) for the central estimate; 0.56 (95% CI 0.33–0.94) using North London and SLSR data only.

The (unadjusted) Kaplan-Meier survival rate at 90 days was 81.5% ‘before’ (95% CI 76.0%–85.9%) and 88.7% ‘after’ (95% CI 87.4%–89.9%), with a 39% reduction in deaths at 90 days (95% CI 11%–58%). The predicted survival rates at 90 days in the deterministic unadjusted model were 85.0% ‘before’ (95% CI 84.5%–85.5%) and 88.7% ‘after’ (95% CI 88.6%–88.8%); a reduction in deaths of 25% (95% CI 21%–28%). Survival at 90 days in the ‘before’ model increased to 87.2% (95% CI 86.7%–87.7%) when the model was adjusted for the decrease in deaths at 30 days seen over the same period in the rest of England, giving a reduction in adjusted deaths of 12% (95% CI 8%–16%).

**Figure 3:**
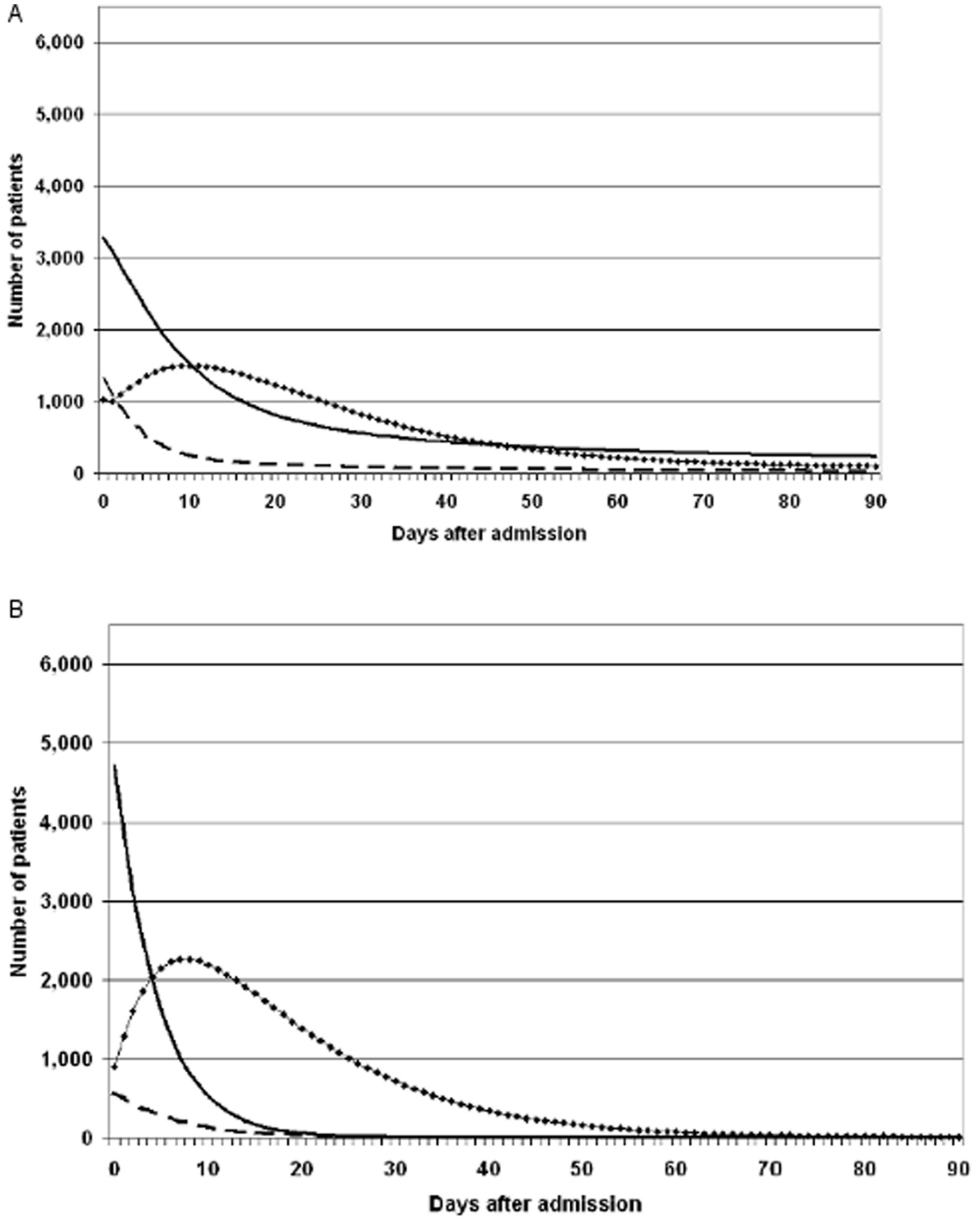
Patients on stroke units and on the medical ward from stroke onset to 90 days after stroke. **A Before period. ———** Acute Stroke Unit. **——•—** Stroke Rehabilitation Unit. **- - - - -** Medical ward. **B After period. ———** Hyper Acute Stroke Unit. **——•—** Stroke Unit. **- - - - -** Medical ward.

Around 50% of patients ‘before’ were admitted initially to an SU and around 10% were in the SU or stroke rehabilitation at 90 days (Figure 3). ‘After’, three-quarters of patients were admitted initially to a HASU and less than 1% were in the HASU or the SU at 90 days. Around 5% of patients were discharged to a nursing home and 60% home at 90 days ‘before’ compared with 1% and 70%, respectively ‘after’ (See Figures S3–S6 in File S1).

Mean ambulance response times and scene-to-hospital times increased in the ‘after’ period. From January 2005–March 2008 the mean time from emergency call to arrival at hospital was 50 minutes and scene-to-hospital time was 14 minutes. From July 2010–May 2011 they were 62 and 17 minutes, respectively.

The rate of intravenous thrombolysis treatment as a percentage of all stroke patients increased from 5% in the ‘before’ period to 12% in the ‘after’ period.

At 90 days the new model is less costly and produces better health outcomes. The 90-day cost per patient to treat a stroke ‘before’ was £14,117 (95% CI £14,092–£14,143; US$22,767 [15]) compared to £13,306 ‘after’ (95% CI £13,286–£13,327; US$21,460). This translates to a 90-day cost saving of £5.2 million (95% CI £4.9–£5.5 million; US$8.4 million) across 6,438 patients in the model (£811 per patient; US$1307).

There were 125 fewer deaths (95% CI 118–132) and 93.59 more QALYs (95% CI 91.82–95.36; 0.015 per patient) during the first 90 days following admission: the new model saved lives and produced more QALYs. Basing the analysis only on the North London and SLSR data, the new model costs £295 (95% CI £242–£347; US$476) more per patient, with an incremental cost per death averted of £16,779 (US$27,066) and cost per QALY gained at 90 days of £56,940 (Table 2).

**Table 2:** Results of cost-effectiveness analysis and sensitivity analysis time horizon 90 days: After minus before.

	Diff. in totalcosts	Diff. in totaldeaths	Inc. cost/deathaverted	Diff. in totalQALYs	Inc. cost/QALYgained
Central estimate (probabilistic)	−5,221,877	−125	Dominant	94	Dominant
North London and SLSR data only(probabilistic)	1,898,440	−113	16,779	33	56,940
Unadjusted for national trends in mortalityand length of stay in strokeunits (probabilistic)	−6,765,485	−254	Dominant	118	Dominant
Adjusted for national trends in mortalitybut not length of stay in stroke units(deterministic)	−7,144,790	−87	Dominant	97	Dominant
Adjusted for national trends in length ofstay in stroke units but notmortality (deterministic)	−1,779,815	−235	Dominant	99	Dominant
Adjustment for stroke mimics (deterministic)	−2,371,637	−81	Dominant	84	Dominant
Reduced length of stay in HASU(deterministic)	−7,776,818	−99	Dominant	89	Dominant
Increase unit cost per day in HASU by 25%(deterministic)	226,268	−98	2,302	86	2,631
Unadjusted length of stay in ICU(deterministic)	−12,508,546	−97	Dominant	90	Dominant
Adjusted neurosurgery rates (deterministic)	−3,544,210	−98	Dominant	86	Dominant
NHS costs only (deterministic)	−1,507,197	−98	Dominant	86	Dominant
Patients in hospital at three monthsdischarged to home (deterministic)	−3,544,210	−98	Dominant	86	Dominant

Total cost, deaths and QALYs calculated over 6438 patients. All costs in 2010/11 UK£ (key figures in US$ in text). In the difference (“Diff.”) columns negative (positive) costs, deaths and QALYs mean that costs, deaths and QALYs are lower (higher) in the After period compared with the Before period. “Dominant” means that costs are lower and either deaths are lower or QALYs are higher in the After period compared with the Before period.

When the model is carried out to ten years, the new model is dominant in every scenario tested. The discounted 10-year cost per patient in the ‘before’ model is £39,614 (95% CI £39,549–£39,678; US$63,895) compared to £35,745 (95% CI £35,697–£35,793; US$57,654) ‘after’. Across 6,438 patients costs are £24.9 million (95% CI £24.1 million–£25.6 million; US$40.2 million) lower ‘after’ compared with ‘before’ and there are 4,193 (95% CI 4166–4221) QALYs gained (0.65 per patient) (Table 3).

**Table 3:** Results of cost-effectiveness analysis and sensitivity analysis time horizon 10 years: After minus before.

	Diff. in total costs	Diff. in total QALYs	Inc. cost/QALY gained
Central estimate (probabilistic)	−24,905,053	4,193	Dominant
North London and SLSR data only (probabilistic)	−2,594,900	2,737	Dominant
Unadjusted for national trends in mortality and length of stay in stroke units (probabilistic)	−23,729,977	4,220	Dominant
Adjusted for national trends in mortality but not length of stay in stroke units (deterministic)	−28,547,614	4,116	Dominant
Adjusted for national trends in length of stay in stroke units butnot mortality (deterministic)	−15,831,855	4,385	Dominant
Adjustment for stroke mimics (deterministic)	−21,831,909	3,978	Dominant
Reduced length of stay in HASU (deterministic)	−27,904,017	4,045	Dominant
Increase unit cost per day in HASU by 25% (deterministic)	−18,109,865	4,035	Dominant
Unadjusted length of stay in ICU (deterministic)	−33,571,242	4,039	Dominant
Adjusted neurosurgery rates (deterministic)	−22,699,835	4,035	Dominant
NHS costs only (deterministic)	191,094	4,035	47
Patients in hospital at three months discharged to home (deterministic)	−14,410,215	4,346	Dominant

Total cost, deaths and QALYs calculated over 6438 patients. All costs in 2010/11 UK£ (key figures in US$ in text). In the 10 year model costs and benefits are discounted at an annual rate of 3.5%. In the difference (“Diff.”) columns negative (positive) costs, deaths and QALYs mean that costs, deaths and QALYs are lower (higher) in the After period compared with the Before period. “Dominant” means that costs are lower and either deaths are lower or QALYs are higher in the After period compared with the Before period.

**Figure 4:**
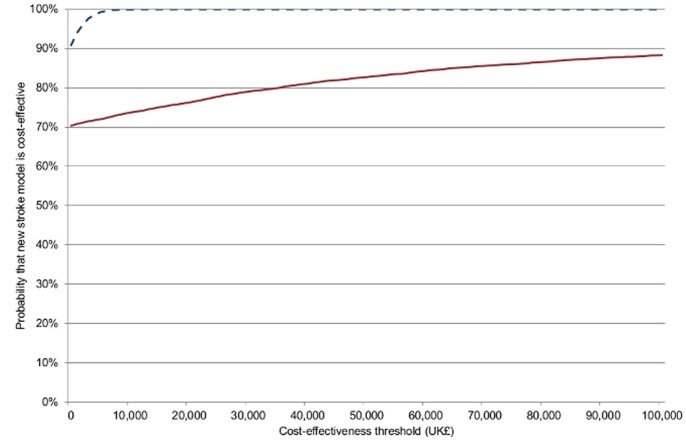
Cost-effectiveness acceptability curves. **- - - - -** 10 years. **———** 90 days. The curves in the figure graph the probability that the new London Stroke Service is cost-effective against the cost-effectiveness threshold measured in terms of the incremental cost per QALY gained. This accounts simultaneously for uncertainty in the cost-effectiveness estimates and in the value of the cost-effectiveness threshold (the level of cost-effectiveness that the new London Stroke Service needs to be more cost-effective than, i.e., have a lower incremental cost per QALY gained than to be considered good value for money). In England the cost-effectiveness threshold used by NICE is in the range £20,000–£30,000 per QALY gained (US$31,000–£46,500 using an exchange rate of UK£1 = US$1.55). Curves are shown for each time horizon.

Sensitivity analyses show that the findings are consistent after testing key assumptions (Table 2–3). See Figure 4 for cost-effectiveness acceptability curves and Figures S7–S8 in File S1 for results of the Monte Carlo.

## Discussion

This study has demonstrated that reconfiguration of acute stroke care across a metropolitan population of 8 million people can achieve improved clinical outcomes while also being cost-effective. Using a 90-day time horizon, the new model costs £811 (US$1307) less per patient, with fewer deaths and improved quality-adjusted survival. Over 10 years, the effects are maintained and the new model costs £3868 (US$6233) less per patient.

Prior to the introduction of the new London acute stroke model, there was marked variation in access to specialised stroke care with just over half of patients being admitted to designated stroke units and only 5% of acute stroke patients receiving thrombolysis treatment. Following the reconfiguration, over 75% of patients received immediate expert assessment and treatment and thrombolysis rates increased to 12%.

Significant financial investments were made to bring about these changes. It has been estimated that a capital investment of around £9 million (US$14 million) was made to meet the requirements for the new HASUs and SUs. [16] If we assume that the consultation and accreditation process costed a further £1 million (US$1.6 million), the total implementation cost would be around £10 million (US$16 million); this investment would be recouped within around two years according to our figures.

This comparative effectiveness research study has provided clinical outcome and cost effectiveness data from a very large and complex clinical service re-organization. The scale of the project and the requirement for rapid and systematic implementation has provided several challenges for this evaluation. We have had to use a variety of data sources and methods to provide results that can be used by patients, clinicians and policymakers.

Our study has several limitations. One is that before-and-after study designs are less robust than randomized control trials (RCT) as the design means less control over confounding variables and sources of bias. It was not possible to use an RCT design given the nature of the intervention being evaluated, i.e., city-wide service reorganization. We tried to account for potential confounders and biases using several independent, population based data sets to model the stroke care pathway in London before and after the introduction of the new model, by being conservative in our methods and by testing our assumptions in sensitivity analyses. We adjusted our analyses to reflect national trends in mortality and LOS, the main drivers of costs and outcomes. Improvements in mortality and LOS that occurred in London between 2007/08 and 2010/11 were greater than that seen by the rest of the country over the same period [5], [10].

It is apparent that mortality from stroke has dropped across the whole of England over the last three years [10]. However the reduction has been disproportionately greater in London following the introduction of the new centralized model. This study also demonstrated a significant increase in thrombolysis rates from 5% to 12% following the reconfiguration. Although the aim to improve thrombolysis rates was an important driver in the development of the new model, it does not fully explain the QALY gains associated with the reconfiguration, given that thrombolysis was still only received by a minority of patients.

An RCT from Australia has demonstrated that the implementation of early, multi-disciplinary supported, evidenced based protocols targeting important areas such as swallowing substantially improve stroke outcomes up to 90 days after admission for stroke [17]. The new model of stroke care we have described benefits from bundles of care delivered by highly specialised nursing, therapy and medical teams who are assessing and treating patients from the time of hospital admission. It is highly probable that the consolidation of expertise and treating higher volumes of patients leads to improved diagnosis and overall improved processes of care. This is more likely to reduce peri-stroke complications and may therefore explain in part the reduced mortality observed in our study.

Our results are also consistent with other studies showing that treating patients in dedicated stroke units is cost-effective, [18], [19] and our calculations of the short- and long-term costs of treating stroke are similar to previous UK studies. [20].

The only measure available to calculate utility scores for QALYs from was the BI. Although the BI is a valid measure of daily living in stroke patients, it is less sensitive to severe and minor stroke events, suffering from ceiling and floor effects. [21] This may have resulted in an underestimation of the total QALYs, with more patients at the higher extremes than at the lower. This would either have equal impact on the ‘before’ and ‘after’ period if there was no difference between the two time periods in severity of stroke, or underestimated the total QALY gain if there was an improvement in functional impairment in the ‘after’ period, as was seen.

Our study shows that a system directing patients to high quality stroke units in the first 72 hours following stroke saves lives and saves money. This was delivered using a centralized model, which worked well in London because of the high density population and hospital distribution that permitted ambulance travel times to remain within viable limits. While our study could be used to support the implementation of consolidated hyper-acute stroke care in other large populations, further research is required to examine whether the London model is viable in other geographical and clinical settings.

## Supporting Information

File S1 Table S1, Main data sources used to model health outcomes and volume of resource use. Methods S1, Further details of short-run model. Methods S2, Further details of long-run model. Methods S3, Deterministic and probabilistic sensitivity analysis. Figure S1, Movement of patients in the short-run cost-effectiveness model from stroke onset to 3 months after stroke onset. Table S2, EQ-5D utility scores and QALYs. Table S3, Unit Costs. Figure S2, Movement of patients in the short-run cost-effectiveness model from 3 months after stroke onset until up to 10 years after stroke onset. Table S4, Transition probabilities in long-run model. Table S5, Barthel Index categories at three months after acute stroke among those at home. Table S6, Parameters and distributions used in the probabilistic sensitivity analysis. Figure S3, Distribution of patients between states from stroke onset to 90 days after stroke: Before period. Figure S4, Distribution of patients between states from stroke onset to 90 days after stroke: After period. Figure S5, Distribution of patients between states from 90 days to ten years after stroke: Before period. Figure S6, Distribution of patients between states from 90 days to ten years after stroke: After period. Figure S7, Monte Carlo simulations of incremental cost per QALY gained of new London stroke service using 90-day time horizon. Figure S8, Monte Carlo simulations of incremental cost per QALY gained of new London stroke service using ten year time horizon.
(PDF)

## References

[1] Mackay J, Mensah G (2004) The atlas of heart disease and stroke. Geneva: World Health Organisation. 112 p.

[2] Stroke Unit Trialists Collaboration (2002) Organised inpatient (stroke unit) care for stroke. Cochrane Database Syst Rev 4: CD000197. 10.1002/14651858.CD000197 11869570

[3] Xian Y, Holloway RG, Chan PS, Noyes K, Shah MN, et al (2011) Association between stroke center hospitalization for acute ischemic stroke and mortality. JAMA 305: 373–380. 21266684 10.1001/jama.2011.22 PMC3290863

[4] Healthcare for London (2007) A Framework For Action. Available: http://www.nhshistory.net/darzilondon.pdf. Accessed 1 July 2013.

[5] Hoffman A, Grant R, Wurie F, Campbell J, Lowe D, et al.. (2009) The National Sentinel Audit of Stroke 2008. London: Royal College of Physicians. 114 p.

[6] Healthcare for London (2008) Stroke strategy for London. Available: http://www.londonhp.nhs.uk/wp-content/uploads/2011/03/London-Stroke-Strategy.pdf. Accessed 1 July 2013.

[7] Healthcare for London (2009) Stroke acute commissioning and tariff guidance. Healthcare for London. Available: http://www.londonhp.nhs.uk/wp-content/uploads/2011/03/Stroke-Commissioning-and-Tariff-Guidance.pdf. Accessed 1 July 2013.

[8] Sarker SJ, Heuschmann PU, Burger I, Wolfe CD, Rudd AG, et al (2008) Predictors of survival after haemorrhagic stroke in a multi-ethnic population: the South London Stroke Register (SLSR). J Neurol Neurosurg Psychiatry 79: 260–265. 18032456 10.1136/jnnp.2007.129189

[9] Wolfe CD, Crichton SL, Heuschmann PU, McKevitt CJ, Toschke AM, et al (2011) Estimates of outcomes up to ten years after stroke: analysis from the prospective South London Stroke Register. PLoS Med 8 e1001033: 10. 10.1371/journal.pmed.1001033 PMC3096613 21610863

[10] Hensage U, Hoffman A, Kavanagh S, Roughton M, Rudd AG, et al.. (2011) The National Sentinel Audit of Stroke 2010. London: Royal College of Physicians. 78 p.

[11] Kaambwa B, Billingham L, Bryan S (2013) Mapping utility scores from the Barthel index. Eur J Health Econ 14: 231–241. 22045272 10.1007/s10198-011-0364-5

[12] Briggs A, Claxton K, Schulper M (2006) Decision Modelling for Health Economic Evaluation. Oxford: Oxford University Press. 256 p.

[13] NHS Information Centre (2011) Emergency hospital admissions: stroke: indirectly standardised rate, all ages, annual trend, P. Available: https://indicators.ic.nhs.uk/download/NCHOD/Data/10C_528ISR7CP_11_V1_D.xls.Accessed 1 July 2013.

[14] National Institute for Health and Clinical Excellence (2008) Guide to the methods of technology appraisal. London: NICE publications. 76 p. 27905712

[15] XE.COM INC (2012) XE Currency Converter. Available: http://www.xe.com/. Accessed 5 October 2012.

[16] Sheehan J (2009) Pre-consultation business case - Major trauma and stroke services in London. London: Healthcare for London. 145 p.

[17] Middleton S, McElduff P, Ward J, Grimshaw JM, Dale S, et al (2011) Implementation of evidence-based treatment protocols to manage fever, hyperglycaemia, and swallowing dysfunction in acute stroke (QASC): a cluster randomised controlled trial. Lancet 378: 1699–1706. 21996470 10.1016/S0140-6736(11)61485-2

[18] National Audit Office (2010) Progress in improving stroke care: report on the findings from our modelling of stroke provision. Available: http://www.nao.org.uk/wp-content/uploads/2010/02/0910291_modelling.pdf. Accessed 1 July 2013.

[19] Saka O, Serra V, Samyshkin Y, McGuire A, Wolfe CC (2009) Cost-effectiveness of stroke unit care followed by early supported discharge. Stroke 40: 24–29. 19008473 10.1161/STROKEAHA.108.518043

[20] Youman P, Wilson K, Harraf F, Kalra L (2003) The economic burden of stroke in the United Kingdom. Pharmacoeconomics 21 Suppl 143–50. 12648034 10.2165/00019053-200321001-00005

[21] Quinn TJ, Langhorne P, Stott DJ (2011) Barthel Index for stroke trials: development, properties and application. Stroke 42: 1146–1151. 21372310 10.1161/STROKEAHA.110.598540

