# Supplementary material for: Impact on Clinical and Cost Outcomes of a Centralized Approach to Acute Stroke Care in London: A Comparative Effectiveness Before and After Model
Source: PLoS One. 2013 Aug 1;8(8):e70420. doi: 10.1371/journal.pone.0070420 (PMC3731285; doi:10.1371/journal.pone.0070420)
Supplement: File S1 — Table S1, Main data sources used to model health outcomes and volume of resource use. Methods S1, Further details of short-run model. Methods S2, Further details of long-run model. Methods S3, Deterministic and probabilistic sensitivity analysis. Figure S1, Movement of patients in the short-run cost-effectiveness model from stroke onset to 3 months after stroke onset. Table S2, EQ-5D utility scores and QALYs. Table S3, Unit Costs. Figure S2, Movement of patients in the short-run cost-effectiveness model from 3 months after stroke onset until up to 10 years after stroke onset. Table S4, Transition probabilities in long-run model. Table S5, Barthel Index categories at three months after acute stroke among those at home. Table S6, Parameters and distributions used in the probabilistic sensitivity analysis. Figure S3, Distribution of patients between states from stroke onset to 90 days after stroke: Before period. Figure S4, Distribution of patients between states from stroke onset to 90 days after stroke: After period. Figure S5, Distribution of patients between states from 90 days to ten years after stroke: Before period. Figure S6, Distribution of patients between states from 90 days to ten years after stroke: After period. Figure S7, Monte Carlo simulations of incremental cost per QALY gained of new London stroke service using 90-day time horizon. Figure S8, Monte Carlo simulations of incremental cost per QALY gained of new London stroke service using ten year time horizon. (PDF) [file pone.0070420.s001.pdf]

Impact on Clinical and Cost Outcomes of a Centralized Approach to Acute Stroke Care in London: A Comparative Effectiveness Before and After Model

Supporting Information

Table S1. Main data sources used to model health outcomes and volume of resource use

Methods S1. Further details of short-run model

Methods S2. Further details of long-run model

Methods S3. Deterministic and probabilistic sensitivity analysis

Figure S1. Movement of patients in the short-run cost-effectiveness model from stroke onset to 3 months after stroke onset

Table S2. EQ-5D utility scores and QALYs

Table S3. Unit Costs

Figure S2. Movement of patients in the short-run cost-effectiveness model from 3 months after stroke onset until up to 10 years after stroke onset

Table S4. Transition probabilities in long-run model

Table S5. Barthel Index categories at three months after acute stroke among those at home

Table S6. Parameters and distributions used in the probabilistic sensitivity analysis

Figure S3. Distribution of patients between states from stroke onset to 90 days after stroke: Before period

Figure S4. Distribution of patients between states from stroke onset to 90 days after stroke: After period

Figure S5. Distribution of patients between states from 90 days to ten years after stroke: Before period

Figure S6. Distribution of patients between states from 90 days to ten years after stroke: After period

Figure S7. Monte Carlo simulations of incremental cost per QALY gained of new London stroke service using 90-day time horizon

Figure S8. Monte Carlo simulations of incremental cost per QALY gained of new London stroke service using ten year time horizon

Main data sources used to model health outcomes and volume of resource use

Table S1. Main data sources

	**South London Stroke Register (SLSR)**	**Stroke Improvement National Audit Programme (SINAP)**	**London Minimum Dataset (LMDS)**	**Sentinel Stroke Audit (SSA)**	**North London dataset**	**London Ambulance Service (LAS)**
Brief description	Prospective stroke register covering a multi-ethnic population of around 240,000 in South London	National clinical audit, focusing particularly on care standards	Dataset for London stroke services to enable reporting against key national and local priorities	National clinical audit occurring every two years to monitor stroke care against national standards	Bespoke retrospectively collected stroke audit data from 2 North London hospitals	Operational data for LAS
Time period	July 2007 to June 2008<br>July 2010 to January 2011	January 2011 onwards	January 2011 onwards	April to June 2008;<br>April to June 2010	April to June 2008;<br>April to June 2011	January 2005 onwards
Part of stroke pathway covered	Stroke onset to discharge from acute care and beyond	First 72 hours after admission	Stroke unit to discharge from acute care	Stroke onset to discharge from acute care	Stroke onset to discharge from acute care	999 call to hospital admission
Patients	All stroke patients in 22 wards in South London accessing 5 hospitals	All new stroke admissions in England – London data collected as part of LMDS only	All stroke patients in London stroke network that pass through dedicated stroke services	All providers of stroke care in England; first 60 consecutive cases	321 consecutive stroke patients at 2 North London Hospitals	All stroke patients that elicit a response from the LAS
Number of observations used in calculations	Before – 205<br><br>After – 100	(See LMDS)	2,837	2008 – 10,077<br><br>2010 – 10,080	Before – 102<br><br>After – 219	30,740
Relevant data	Mortality, Barthel Index, length of stay, imaging and interventions, staff contacts, drug use, post discharge destinations	Mortality, Barthel Index	Length of stay, Barthel Index	Comparisons between London and rest of England; validation for 2008 data	Mortality, Barthel Index, length of stay, imaging and interventions, staff contacts, drug use, post discharge destinations	Ambulance journey times

Methods S1. Further details of short-run model

Model structure

The short-run model has a time horizon of three months (90 days). The model accounts for ambulance travel time to hospital admission, time spent in hospital and, for patients who are discharged before three months, the time after hospital discharge.

The outcome measures are deaths averted and QALYs gained (positive or negative) in the After period compared with the Before period and the cost-effectiveness measures are the incremental cost per death averted and the incremental cost per QALY gained at 30 days and at three months.

For the ambulance travel time to hospital admission we use data provided by LAS. For the Before period we obtained data on ambulance response times in London from January 2005 to March 2008 for 23,365 stroke patients, defined according to LAS illness code 39 ("Neurological / CVA") who were conveyed to hospital via LAS. For the After period we obtained data for 7375 'Face Arms Speech Time' (FAST) positive patients conveyed directly to a HASU or to A&E in London by LAS over the period July 2010 to May 2011. Note that in the Before period the stroke patients included in the sample may not have been FAST positive because this was not a routine test during that period. From these data we extracted information on the mean time in minutes from 999 call to the arrival of the ambulance at the scene, the mean time in minutes spent by the ambulance at the scene, and the mean journey time from the scene to the hospital. We also extracted data on the time spent conveying stroke patients from the A&E to a HASU. Not all patients arriving at hospital with acute stroke travelled to hospital via LAS, and we account for the cost implications of this in our model.

To model the time spent in hospital, and after hospital discharge within the three month time horizon, we use a Markov model structure, with cycle length of one day (Figure S1). The model consists of 90 one-day cycles, and models the movement of acute stroke patients through the stroke pathway from arrival at hospital to three months after stroke onset.

In the Before period, on arrival at the hospital patients are admitted to one of the following locations (the initial states in the model): ASU; stroke rehabilitation; medical ward; surgical ward; ICU; hospitalisation - other. In subsequent cycles patients may be in one of the following states:

- Acute hospitalisation:
    - ASU
    - Stroke rehabilitation
    - Medical ward
    - Surgical ward
    - ICU
    - Hospitalisation - other
- Post discharge care:
    - Nursing home
    - Community hospital
    - Inpatient rehabilitation
    - Acute hospital transfer
    - Discharge - other
- Home
- Dead

In the After period patients are admitted to one of the following: HASU; SU; medical ward; surgical ward; ICU; hospitalisation - other. In subsequent cycles they may be in one of the following states:

- Acute hospitalisation:
    - HASU
    - SU
    - Medical ward
    - Surgical ward
    - ICU
    - Hospitalisation - other
- Post discharge care:
    - Nursing home
    - Community hospital

    - Inpatient rehabilitation
    - Acute hospital transfer
    - Discharge - other
- Home
- Dead

In both the Before and After periods the following transitions between states are possible:

- Patients in any of the acute hospitalisation states can be admitted to any one of the other acute hospitalisation states, can be discharged to any of the post discharge care locations, can be discharged to home, can die, or can remain in their current state.
- Patients in any of the post discharge care locations can die or can remain in their current state.
- Patients discharged to home can die or can remain in their current state.

We assume that once patients are discharged from acute hospitalisation they remain in the following state (post discharge care, home, dead) until the end of the three month period, and do not move between these states unless they die. Evidence shows that this is a reasonable representation of reality, e.g., there are examples of patients typically being admitted to inpatient rehabilitation for eight to 12 weeks after acute hospitalisation[1], and recommendations are that patients admitted to community hospitals for specialist stroke rehabilitation ought to remain there for 28 days[2]. We investigated the importance of this assumption in a sensitivity analysis, examining what would happen if all those in post-discharge care were instead discharged to home from acute hospitalisation and did not move out of this state unless they died.

Transition probabilities

The admission source and the first location, i.e., the initial distribution of patients between states in the Markov model, were obtained from the SLSR, North London dataset for the Before period and from these sources plus the LMDS in the After period.

Subsequent movements were based on one-day time dependent transition probabilities. Time dependence was measured in terms of the time from admission to the first location. The transition probabilities were derived as follows:

- For each initial location we model 'time to event' where the event is movement out of that location. The appropriate way of analysing 'time to event' data is using survival analysis. We use the parametric Weibull model, and run separate survival models for each ward transfer, modelling time to event for each of the possible initial locations in the Before and After periods. In the survival models we include variables denoting the type of location discharged to, retaining those that were statistically significant. The data used in these analyses were patient level data from the SLSR and North London data for the Before period and from these sources plus the LMDS for the After period. Patients are only included in the models if they have a date of initial hospital admission and date of admission to the next ward or date of discharge.
- The hazard ratios produced by these models were converted into one day transition probabilities using methodology provided by Briggs et al[3]. The transition probabilities give the probability that the patient will leave the initial location each day. They are time dependent in that the transition probability varies by the number of days that have passed since admission to the first ward and the type of location discharged to, where this was statistically significant in the survival model.
- Movements for patients with a length of stay less than one day, i.e., patients that are admitted and discharged on the same day were calculated separately. For the first ward patients were admitted to, we calculated the percentage of patients for each ward type that had a length of stay less than one day. Of those patients we calculated the proportion discharged to each location.
- We combine these data with discharge destination data from the same sources used to model the time to event data. These data measure the proportion of patients discharged to each location (acute hospitalisation, post-discharge care, or home). These values are not time dependent, but are based specifically on the next destination in the care pathway.
- We then combine the transition probabilities and the discharge destination data to calculate the probability that patients will leave each initial location each day, and the probability of the new location they will then move to when they do leave.
- For second and following locations we model the number of patients moving into each new location each day.

- We then model movements out of this location by repeating the methods described above, where time to event in the survival models is the time from the initial hospital admission to the movement out of the second location. The discharge destination data are based on the third destinations in the care pathway.
- We repeat this process for up to four movements over the 90 day period.
- The probability of dying on each day is run as a separate survival model using the same mortality rate across all locations to model the probability of a death on each day directly. For the survival model, date of entry is the date admitted to the first ward. For the North London dataset death was only reported if it occurred while the patient was still in hospital. For SLSR and LMDS follow-up was until the last date that the dataset had been updated for death data, which is obtained from ONS. Patients with no initial hospital admission date were not included in the model.
- The model includes admissions to the ICU. It is possible that other policy initiatives unrelated to the London Stroke Strategy may have have contributed to a reduced length of stay on the ICU in the After period. As a result, in the Before model, although we left the percentage of patients entering the ICU as a first, second or third ward the same as that indicated by the data, all patients entering an ICU as a fourth ward in the Before period were discharged to inpatient rehabilitation instead. The length of stay was also reduced for all patients that were admitted to the ICU as a third ward so that the number of patients with an extended length of stay in the ICU was as close as possible in the Before period as the After period. This was done by amending the constant term in the Weibull model.

Measuring EQ-5D utility scores and QALYs

EQ-5D utility scores during acute hospitalisation are calculated based on Barthel Index (BI) scores measured during the first seven days after stroke onset by location (ASU, stroke rehabilitation, HASU, SU, medical ward, surgical ward, ICU, hospitalisation - other). BI scores were obtained from the SLSR, and North London data. We assume that BI scores are the same for patients on the medical ward, surgical ward, ICU or hospitalisation - other in both the Before and After periods and calculate the BI scores for these locations using data pooled across patients in both periods. The BI scores were converted to EQ-5D utility scores using a new UK-based algorithm developed by Kaambwa et al[4].

For patients in post-discharge care and patients at home we used the same procedure, using BI scores measured at different locations at discharge for the North London dataset and at three months after stroke onset using SLSR data.

Mean EQ-5D utility scores for each location were converted to daily QALYs by dividing the value by 365, and then applied to each location and day throughout the three month period. The values used in the model are in Table S2.

Measuring costs

The following cost components are included in the short-run model:

- Transport
  - Ambulance journey times
  - Transport from HASU to SU
- Acute hospitalisation
  - HASU
  - ASU
  - SU
  - Rehabilitation unit
  - Medical ward
  - Surgical ward
  - ICU
  - Hospitalisation - other
- Imaging and surgical interventions
  - Head CT scan
  - Head MRI scan
  - Angiogram
  - ECG
  - Echocardiogram
  - Catheter and carotid stenting

    - Neurosurgery
- Staff contacts
    - Stroke specialist physician/consultant
    - Occupational therapist
    - Physiotherapist
    - Speech and language therapist
    - Psychologist
    - Dietician
    - Social worker
- Medications during acute hospitalisation
    - Thrombolysis
    - Warfarin
    - Antiplatelets
    - Statins
- Post-discharge care
    - Nursing home
    - Community hospital
    - Inpatient rehabilitation
    - Acute hospital transfer
    - Discharge - other
    - Early supported discharge
    - Community rehabilitation
    - Post discharge medications

Volume of resource use data for each of these cost components were assembled from the following sources:

- Ambulance journey times were obtained from the LAS using the methods described above.
- Transportation of patients from the HASU to the SU in the After period were based on estimates that 36% of SU beds that are located in the same hospital as a HASU. We assumed that similar costs were not incurred in the Before period.
- The number of days in each location during acute hospitalisation (ASU, stroke rehabilitation, HASU, SU, medical ward, surgical ward, ICU, other) and post-discharge care (nursing home, community hospital, inpatient rehabilitation, acute hospital transfer, discharge other) was calculated internally by the short-run model.
- Use of imaging and surgical interventions, staff contacts, medications during acute hospitalisation and other components of post-discharge care were based on the SLSR, North London dataset for the Before period and from these data plus the LMDS for surgical interventions and staff contacts during the first 72 hours whilst in the HASU in the After period.

Unit costs for each of these cost components were obtained from published figures and then applied to the volume of resource use data. The values used in the model are in Table S3.

We did not account separately for the costs of nursing staff and junior doctors, since we assumed these costs were included in the daily unit costs for each location.

As the cycle length in the model is one day, ward bed day cost could be calculated for whole days only. As a result, the costs of being on a ward for one day were calculated for all patients who were on the ward at the end of a cycle with the assumption that they had been there for the full 24 hours. Patients that were on a ward for less than a full cycle and ended up in a different location at the end of the cycle did not accrue the costs of being on that ward, but only of the location they were in at the end of that cycle.

The locations ‘hospitalization – other’, ‘acute hospital transfer’ and ‘discharge – other’ were undefined in the data, but combined they accounted for six percent of the distribution of all patients across the whole three month period in both the Before and After periods. We calculate transition probabilities and EQ-5D utility scores separately for these three states. In terms of unit costs, for ‘hospitalization – other’ and ‘acute hospital transfer’ we used the mean of the unit cost per day on the medical ward and surgical ward. For ‘discharge – other’ we used the mean of the unit costs per day of the other post-discharge locations (nursing home, community hospital, inpatient rehabilitation).

Stroke mimics

The data used in our study are potentially contaminated by the presence of stroke mimics. These are patients with non-stroke conditions that present with stroke-like symptoms. Until they are identified as such, stroke mimics will receive the same care as true stroke patients. Once they are identified they are usually transferred to a medical ward outside of the stroke pathway. They may be less severely ill than true stroke patients, and they may have better outcomes and shorter lengths of stay resulting in lower costs. If these patients are not identified until after they have been entered into the datasets we are using then they may affect our cost-effectiveness estimates. If they are more prevalent in our data for the After period this may make the new London stroke service appear more cost-effective than it actually is. Our analysis is based primarily on data from the SLSR, the 2 North London hospitals in the Before period and these datasets plus the LMDS in the After period. There are no stroke mimics in the data from the SLSR and North London data because entry into these data is based on confirmed stroke diagnoses at the end of the acute pathway. In the LMDS an initial stroke diagnosis is made soon after admission following clinical assessments and routine scans. Hence, while more easily identifiable stroke mimics are identified and excluded from the LMDS a small number of difficult to identify stroke mimics may be included. Unfortunately, it is not possible to identify these stroke mimics in the LMDS data. This means that the exact number is unknown and it is not possible to assess their treatment costs and health outcomes. It is also not possible to easily remove these patients from our data. In the absence of data we assume in our central estimate that there are not stroke mimics in the LMDS. In a sensitivity analysis we investigate assume that 5% of patients in our data in the After period were stroke mimics, and 0% of patients in the Before period were stroke mimics, and assume that these patients would have a shorter length of stay than stroke patients, and lower mortality. Hence, when these patients are removed from the model the costs in the After period increase and health outcomes decline.

Measuring cost-effectiveness

The transition probabilities in the model were used to calculate the numbers of patients at each location during the three month time horizon of the short-run model. Deaths at 30 days and at three months were modelled directly in the time to event analyses. Total QALYs were calculated by multiplying the number of patients in each state on each day by the calculated QALYs for that state. Costs associated with acute hospitalisation and location of post-discharge care were calculated in the same way. These were supplemented with total costs for the other cost components included in the analysis, calculated by multiplying the volume of resource use across all patients by the unit cost. Separate calculations were made for the Before and After periods.

Cost-effectiveness was measured as the incremental cost per death averted at 30 days after stroke onset of stroke care in the After period versus stroke care in the Before period, the incremental cost per QALY gained at 30 days after stroke onset, the incremental cost per death averted at three months after stroke onset and the incremental cost per QALY gained at three months after stroke onset. These were calculated as the difference in total costs in the After period and the Before period (the incremental cost) divided by the difference in total benefits (deaths and QALYs, the incremental effectiveness).

Methods S2. Further details of long-run model

Model structure

The long-run model has a time horizon of ten years. The outcome measure is QALYs gained (positive or negative) in the After period compared with the Before period and the cost-effectiveness measure is the incremental cost per QALY gained.

We use a Markov model structure, with cycle length three months (Figure S2). The model consists of 40 three month cycles. The first three month cycle is accounted for by the costs and QALYs in the short-run model. At the end of this first three month period patients are in one of three states: at home; in institutional care (a nursing or residential home); or, dead. Patients at home may be in one of five function levels based on BI scores: Independent (BI score = 20); Mild (BI = 15-19); Moderate (BI = 10-14); Severe (BI = 5-9); or, Very Severe (BI = 0-4). In subsequent cycles patients may be in one of the following states:

- At home (divided into five function levels: Independent, BI score = 20; Mild, BI = 15-19; Moderate, BI = 10-14; Severe, BI = 5-9; or, Very Severe, BI = 0-4);
- In institutional care (a nursing or residential home);
- Recurrent stroke; or
- Dead.

The following transitions between these states are included in the model:

- Patients at home can be admitted to institutional care, can be admitted to hospital for a recurrent stroke, can die, or can remain at home;
- Patients in institutional care can be admitted to hospital for a recurrent stroke, can die, or can remain in institutional care.
- Patients admitted to hospital for a recurrent stroke can be discharged to home, discharged to institutional care, or can die.

We assume that once patients are admitted to institutional care they cannot be discharged to home. Patients at home are divided into five function levels based on BI scores, described above. We also assume that patients do not change their function level unless they have a recurrent stroke.

Transition probabilities

Three-month transition probabilities are either obtained from published studies, from the SLSR, or are calculated internally from the short-run model (Table S4). They were derived as follows:

- At the end of the first three month period comprising the short-run model patients are in one of three states: at home; in institutional care (nursing or residential home); or, dead. The probabilities of being in each of these states were computed internally by the short run model. At the end of the three month period we assumed that half of those still in hospital were discharged to home and half were discharged to institutional care. We investigated the importance of this assumption in a sensitivity analysis, examining what would happen if at three months all those still in hospital were discharged to home and all those in hospital were discharged to institutional care. Among those discharged to home, the probability of being at each function level was based on BI data taken from the SLSR at 3 months after stroke among patients discharged to home (Table S5).
- The probability of moving from home to institutional care was taken from Scott et al[5], based on one year transition probabilities from home into institutional care among people who were hospital inpatients in the previous 12 months.
- The probability of being admitted to a hospital for a recurrent stroke was taken from Mohan et al[6], and based on a meta-analysis of 16 studies. Separate probabilities were extracted for one year, two to five years and six to ten years after the initial stroke. We assume that the probabilities of recurrent stroke are equal for those living at home and in institutional care.
- The probability of dying among those at home was taken from Wolfe et al[7], based on SLSR data. Separate probabilities were extracted for one year, two to five years and six to ten years after the initial stroke.
- The probability of dying among those in institutional care was taken from raw unadjusted data in Bebbington et al[8].
- Following a recurrent stroke, the probability of being discharged to home, discharged to institutional care, or dying was taken from the short-run model. We assume that the probability of each of these

events is the same as for the acute stroke included in the short-run model. As before, we assume that the end of the three month period in the short-run model half of those still in hospital were discharged to home and half were discharged to institutional care. Among those discharged to home, the probability of being at each function level was based on BI data taken from the SLSR at 3 months after stroke among patients discharged to home.

Where transition probabilities were extracted for a period other than three months, these were transformed into three month values using formulae provided by Briggs et al[3].

Measuring EQ-5D utility scores and QALYs

EQ-5D utility scores are calculated for each state in the Markov model based on BI scores at three months by location (at home, institutional care, recurrent stroke). For those at home, utility scores were calculated by function levels (Independent, BI score = 20; Mild, BI = 15-19; Moderate, BI = 10-14; Severe, BI = 5-9; or, Very Severe, BI = 0-4). The BI scores were taken from the SLSR, measured at three months after stroke. We used the algorithm in Kaambwa et al[4] to compute EQ-5D utility scores from the BI scores (Table S2). For recurrent stroke we used separate scores for the Before and After periods calculated by the short-run model.

QALYs for each three month period in each state were calculated by dividing the EQ-5D utility score associated with that state by four. The values used in the model are in Table S2.

Measuring costs

Three month costs in each state in the Markov model were obtained from published studies, or calculated internally by the model. They were calculated as follows:

- Costs for patients at home were taken from Jones et al[9], based on annual costs for disabled and non-disabled stroke patients at home. Following the categorization used by Jones et al[9] those with a BI score in the range 0-9 were counted as disabled and those with a score in the range 10–20 were counted as non-disabled. The annual costs were converted to three month figures by dividing by four.
- Costs for patients in institutional care were taken from Youman et al[10].
- For the costs of recurrent stroke we used the costs calculated for acute stroke in the short-run model. We used separate costs for the Before and After periods.

The values used in the model are in Table S3.

Measuring cost-effectiveness

The transition probabilities in the model were used to calculate the numbers of patients at home (by function level), in institutional care, in hospital with recurrent stroke or dead in each of the three month cycles following an acute stroke up to the time horizon of ten years. Total QALYs and costs were calculated by multiplying the number of patients in each state by the calculated QALYs and costs for that state. Separate calculations were made for the Before and After periods. All costs and benefits in the model after the first year are discounted at an annual rate of 3.5%. Cost-effectiveness was measured in terms of the incremental cost per QALY gained of stroke care in the After period versus stroke care in the Before period. This was calculated as the difference in discounted total costs in the After period and the Before period (the incremental cost) divided by the difference in discounted total QALYs (the incremental effectiveness).

Methods S3. Deterministic and probabilistic sensitivity analysis

We run a series of deterministic sensitivity analyses to investigate the sensitivity of our central estimates to assumptions made:

1. We calculate the incremental cost-effectiveness ratios without adjusting for national trends (outside of London) in mortality and length of stay, and also adjusting only for mortality (length of stay) and not length of stay (mortality).
2. We investigated the impact of stroke mimics by assuming that 5% of patients in our data in the After period were stroke mimics, and 0% of patients in the Before period were stroke mimics. Assuming these patients would have a shorter length of stay than stroke patients, we removed the 5% of patients in our data in the After period with the shortest length of stay. This had the effect of increasing the mean length of stay in the After period by 5%. We therefore re-estimated the results assuming that length of stay in the After period increased by 5% compared with the central estimate to account for the potential impact of stroke mimics. To account for the impact on outcomes, we simultaneously increased the number of deaths in the After period by 2.5% (i.e., multiplied the number of deaths in the After period in the central estimate by 1.025) to reflect worse outcomes of stroke patients compared with stroke mimics.
3. In our central estimate the combined data from the SLSR, North London dataset plus the LMDS suggest that the mean length of stay in the HASU was 4.1 days. This is slightly higher than 4th Quarter figures calculated by North West London Cardiovascular and Stroke Network (3.7 days). Hence, we re-estimated the cost-effectiveness of the new London model using the North West London CardioVascular and Stroke Network mean values.
4. Our estimates of the unit cost per day in the HASU were taken from a report published by the National Audit Office[11]. We investigated the sensitivity of our findings to this value by increasing the unit cost per day in the HASU by 25%.
5. In the short-run model we assume that once patients are discharged from acute hospitalisation they remain in the following state (post discharge care, home, dead) until the end of the three month period, and do not move between these states unless they die. We examined what would happen if all those in post-discharge care were instead discharged to home directly from acute hospitalisation and did not move out of this state unless they died.
6. As described, the number of patients with extended lengths of stay in the ICU in the Before period was changed to be the same as the After period as policy changes to reduce length of stay in ICUs are a potential confounding factor in the model. Another version of the model was run using the Before period values without any manipulation so that there are an increased number of patients with an extended length of stay in the ICU in the Before period.
7. The percentage of patients that received neurosurgery was based on data from the SLSR and North London dataset in the Before period and these datasets plus the LMDS in the After period. Our central estimates, based on these datasets, show a decrease in the percentage of patients receiving neurosurgery, from 6.0% in the Before period to 1.2% in the After period. There may be differences in how neurosurgery is reported in the LMDS compared with the other datasets, in which case these neurosurgery rates may not reflect the true difference between the Before and After periods. We therefore recalculated the results based on neurosurgery rates based on data from the SLSR and North London data only. This increased the percentage of patients receiving neurosurgery in the After period from 1.2% to 3.7%.
8. We calculated the incremental cost-effectiveness ratios taking an NHS only perspective, whereas in the central estimate the perspective is an NHS and Personal Social Services.
9. In the long-run model we assume that at the end of the first three month period patients are in one of three states: at home; in institutional care (nursing or residential home); or, dead. The probabilities of being in each of these states were computed internally by the short run model. At the end of the three month period in the short-run model we assumed that half of those still in hospital were discharged to home and half were discharged to institutional care. We investigated what would happen if at three months, in both the Before and After periods, all those still in hospital were discharged to home and all those in hospital were discharged to institutional care.

Probabilistic sensitivity analysis was undertaken to determine the impact of the uncertainty surrounding the model input parameters used to costs and outcomes. The analysis was based on the incremental cost per QALY gained cost-effectiveness measure at 30 days, 90 days and ten years after stroke onset.

In probabilistic sensitivity analysis, each model parameter is assigned a probability distribution reflecting the amount and pattern of its variation, and cost-effectiveness results are calculated by simultaneously selecting random values from each distribution. The process is repeated 10,000 times in a Monte Carlo simulation of the model to give an indication of how variation in the model parameters leads to variation in the incremental cost per QALY gained for a given combination of parameter values.

Our probabilistic sensitivity analysis accounted simultaneously for uncertainty in the following model parameters:

- Movements between locations during acute hospitalization.
- Probability of death.
- Unit costs.
- Use of ambulance journeys, imaging and surgical interventions, and medications during acute hospitalisation.
- The QALYs associated with each location or state in the short-run and long-run models.

Model parameter values for movements between locations during acute hospitalization were simulated using a Dirichlet distribution to account for the multinomial nature of these data. Parameter values for unit costs were simulated using Gamma distributions. Use of ambulance journeys, imaging and surgical interventions, and medications during acute hospitalisation are measured as proportions, and so the parameter values were simulated using Beta distributions. QALYs were simulated using Gamma distributions and calculated as a utility decrement (1-utility) to allow for negative utilities. The parameters and distributions used in the probabilistic sensitivity analysis are summarised in Table S6.

For movements between locations during acute hospitalization, use of ambulance journeys, imaging and surgical interventions, and medications during acute hospitalisation, and the QALYs associated with each location or state the parameters required for each probability distribution were obtained from the original published sources, or were calculated internally by the short-run and long-run models. For the unit costs only point estimates were available. For the purposes of the probabilistic sensitivity analysis we therefore calculated standard errors around the point estimates assuming upper and lower confidence limits that were 30% higher and 30% lower than the point estimates, respectively.

Results of the probabilistic sensitivity analysis are presented as points on the cost-effectiveness plane and as cost-effectiveness acceptability curves. The latter were based on the proportion of the Monte Carlo simulations that had positive net benefit values as the cost-effectiveness threshold was increased from £0-100,000.

Figure S1. Movement of patients in the short-run cost-effectiveness model from stroke onset to 3 months after stroke onset

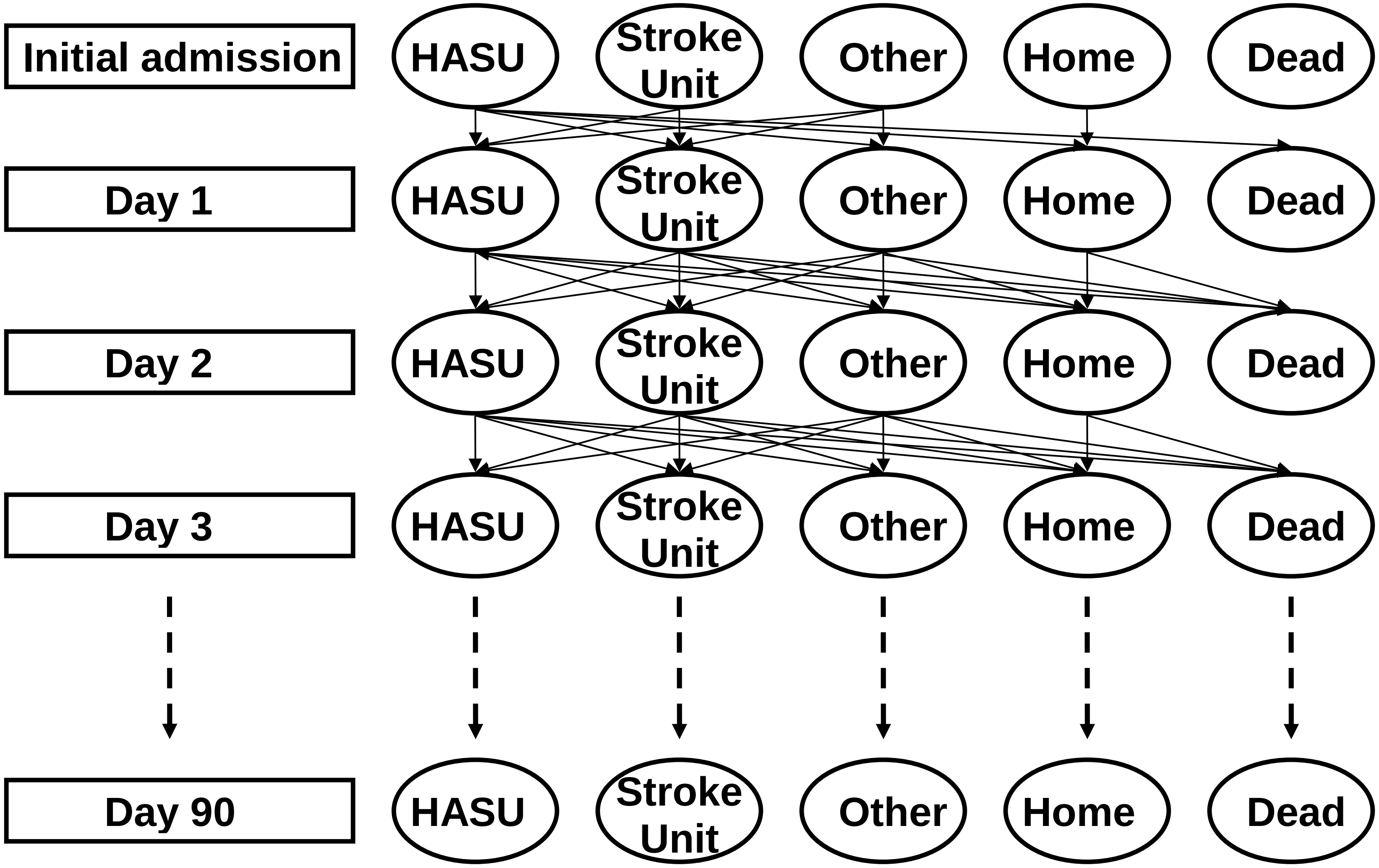

Table S2. EQ-5D utility scores and QALYs

Short-run model				
	Before period		After period	
	EQ-5D utility score	QALYs (over a one day period)	EQ-5D utility score	QALYs (over a one day period)
**Hospitalization location**				
HASU			0.291	0.0008
Acute SU	0.239	0.0007		
SU			0.392	0.0011
Stroke rehabilitation	0.255	0.0007		
	**EQ-5D utility score**		**QALYs (over a one day period)**	
Medical ward	0.266		0.0007	
ICU	0.017		0.00005	
Surgical ward	0.338		0.0009	
Other	0.272		0.0007	
**Post-discharge care location**	**EQ-5D utility score**		**QALYs (over a one day period)**	
Community Hospital	0.159		0.0004	
Inpatient rehabilitation	0.153		0.0004	
Nursing home	0.129		0.0003	
Home	0.558		0.0015	
Acute Hospital Transfer	0.290		0.0008	
Other	0.325		0.0009	
**Long-run model**				
**State**	**EQ-5D utility score**		**QALYs (over a three month period)**	
Home: BI = 20	0.693		0.173	
Home: BI = 15-19	0.595		0.149	
Home: BI = 10-14	0.366		0.092	
Home: BI = 5-9	0.099		0.025	
Home: BI = 0-4	-0.062		-0.016	
Residential Care	0.094		0.024	
Recurrent stroke (Before period)	0.358		0.090	
Recurrent stroke (After period)	0.427		0.107	

Table S3. Unit costs

Cost component	Unit cost (£)	Unit	Source
**From stroke onset to three months after stroke**			
**Transport**			
Ambulance journey times	7.41	Per minute	Curtis[12]
Transport from HASU to SU	41	Per journey	National Reference Costs 2009/10[13]
**Acute hospitalisation**			
HASU	603	Per day	National Audit Office[11]
ASU	317	Per day	Kalra et al.[14]
SU	239	Per day	National Audit Office[11]
Stroke rehabilitation	231	Per day	Kalra et al.[14]
Medical ward	187	Per day	National Audit Office[11]
Surgical ward	211	Per day	Kalra et al.[14], National Audit Office[11]
ICU	1578	Per day	Ridley and Morris[15]
**Imaging and surgical interventions**			
Head CT scan	118	Per scan	National Reference Costs 2009/10[13]
Head MRI scan	141	Per scan	National Reference Costs 2009/10[13]
Angiogram	344	Per test	Mowatt et al.[16]
ECG	71	Per test	Mowatt et al.[16]
Echocardiogram	96	Per test	National Reference Costs 2009/10[13]
Catheter and carotid stenting	3118	Per procedure	National Reference Costs 2009/10[13]
Neurosurgery	6884	Per procedure	National Reference Costs 2009/10[13]
**Staff contacts**			
Stroke specialist physician/consultant	172	Per consultation	Curtis[17]
Occupational therapist	44	Per consultation	Curtis[17]
Physiotherapist	41	Per consultation	Curtis[17]
Speech and language therapist	43	Per consultation	Curtis[17]
Psychologist	82	Per consultation	Curtis[17]
Dietician	32	Per consultation	Curtis[17]
Social worker	70	Per consultation	Curtis[17]
**Medications during acute hospitalisation**			
Thrombolysis	714	Per course of treatment	National Audit Office[11]
Warfarin	0.03	Per day	British National Formulary[18]
Antiplatelets	0.08	Per day	British National Formulary[18]
Statins	0.04	Per day	British National Formulary[18]
**Post discharge care**			
Nursing home	105	Per day	Curtis[17]
Community hospital	105	Per day	Curtis[17]
Inpatient rehabilitation	231	Per day	Kalra et al.[14]
Early supported discharge	158	Per day	Saka et al. (2009)
Community rehabilitation	15	Per day	Kalra et al.[14]
Post discharge medications (Before period)	1.65	Per day	South London Stroke Register, North London dataset, British National

Cost component	Unit cost (£)	Unit	Source
			Formulary[18]
Post discharge medications (After period)	1.83	Per day	South London Stroke Register, North London dataset, British National Formulary[18]
**From three months after stroke to ten years after stroke**			
Ongoing care at home if disabled	1338	Per three-month period	Jones et al.[9]
Ongoing care at home if not disabled	459	Per three-month period	Jones et al.[9]
Ongoing care in an institution	5177	Per three-month period	Youman et al.[10]
Recurrent stroke (Before period)	13827	Per three-month period	Calculated internally by short-run model
Recurrent stroke (After period)	12990	Per three-month period	Calculated internally by short-run model

All costs in 2010/11 UK£

Figure S2. Movement of patients in the short-run cost-effectiveness model from 3 months after stroke onset until up to 10 years after stroke onset

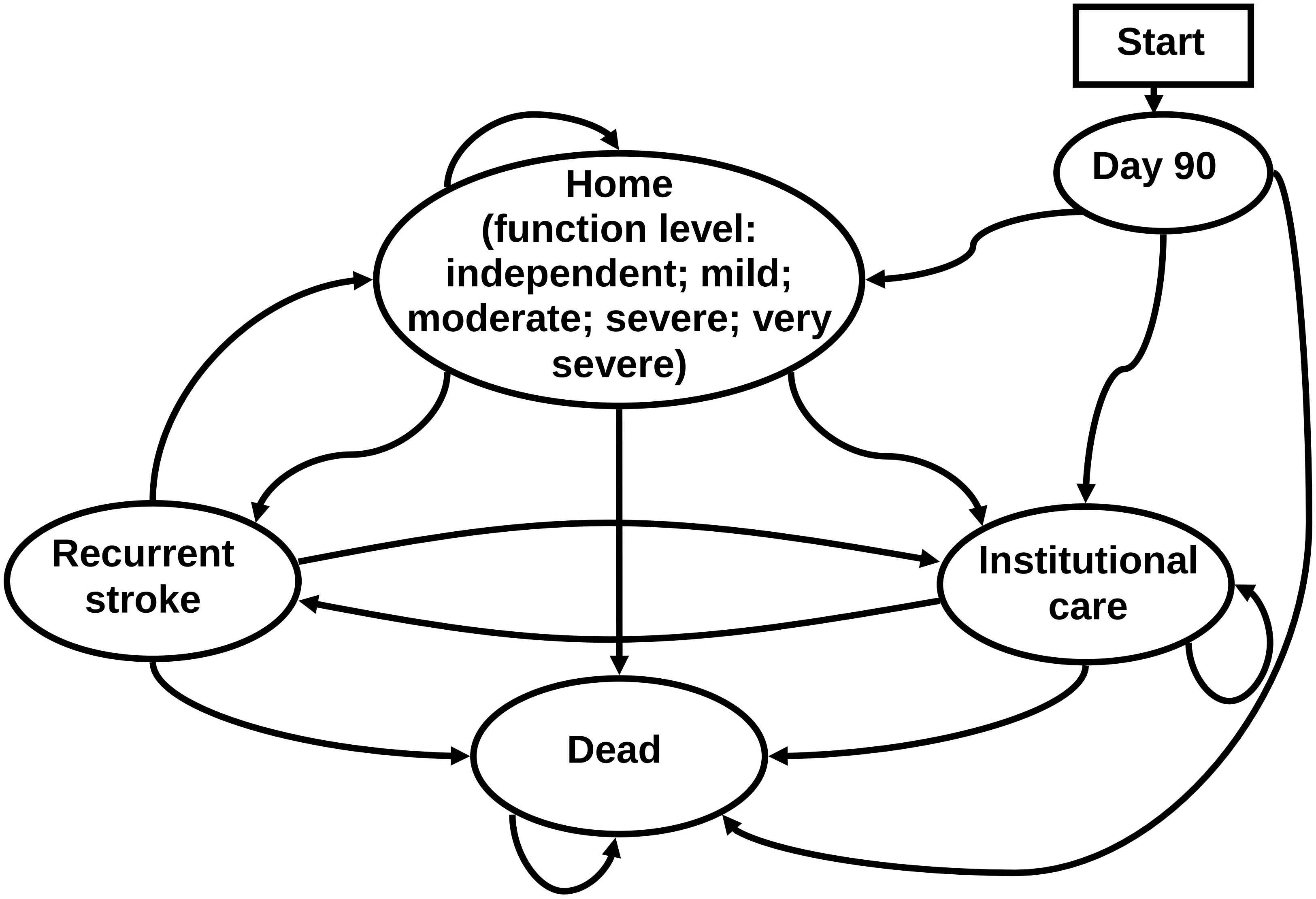

Table S4. Transition probabilities in long-run model

Movement from	Movement to	Three-month Transition probability	Source
Home	Institutional care	0.006	Scott et al[5]
Home	Recurrent stroke (up to end of year 1)	0.029	Mohan et al[6]
Home	Recurrent stroke (years 2 to 5)	0.010	Mohan et al[6]
Home	Recurrent stroke (years 6 to 10)	0.007	Mohan et al[6]
Home	Death (up to end of year 1)	0.030	Wolfe et al[7]
Home	Death (years 2 to 5)	0.014	Wolfe et al[7]
Home	Death (years 6 to 10)	0.010	Wolfe et al[7]
Institutional care	Recurrent stroke (up to end of year 1)	0.029	Mohan et al[6]
Institutional care	Recurrent stroke (years 2 to 5)	0.010	Mohan et al[6]
Institutional care	Recurrent stroke (years 6 to 10)	0.007	Mohan et al[6]
Residential care	Death	0.094	Bebbington et al[8]
Recurrent stroke	Home: BI = 20 (Before period)	0.243	Calculated internally by short-run model, SLSR
Recurrent stroke	Home: BI = 20 (After period)	0.427	Calculated internally by short-run model, SLSR
Recurrent stroke	Home: BI = 15-19 (Before period)	0.197	Calculated internally by short-run model, SLSR
Recurrent stroke	Home: BI = 15-19 (After period)	0.222	Calculated internally by short-run model, SLSR
Recurrent stroke	Home: BI = 10-14 (Before period)	0.106	Calculated internally by short-run model, SLSR
Recurrent stroke	Home: BI = 10-14 (After period)	0.041	Calculated internally by short-run model, SLSR
Recurrent stroke	Home: BI = 5-9 (Before period)	0.061	Calculated internally by short-run model, SLSR
Recurrent stroke	Home: BI = 5-9 (After period)	0.049	Calculated internally by short-run model, SLSR
Recurrent stroke	Home: BI = 0-4 (Before period)	0.076	Calculated internally by short-run model, SLSR
Recurrent stroke	Home: BI = 0-4 (After period)	0.049	Calculated internally by short-run model, SLSR
Recurrent stroke	Institutional care (Before period)	0.173	Calculated internally by short-run model
Recurrent stroke	Institutional care (After period)	0.098	Calculated internally by short-run model
Recurrent stroke	Dead (Before period)	0.145	Calculated internally by short-run model
Recurrent stroke	Dead (After period)	0.113	Calculated internally by short-run model

Table S5. Barthel Index categories at three months after acute stroke among those at home

Category	BI score	Categories used by Jones et al (2004)	Before period		After period	
			Observations	Proportion	Observations	Proportion
Independent	20	Non-disabled	16	0.356	52	0.542
Mild	15-19	Non-disabled	13	0.289	27	0.281
Moderate	10-14	Non-disabled	7	0.156	5	0.052
Severe	5-9	Disabled	4	0.089	6	0.063
Very Severe	0-4	Disabled	5	0.111	6	0.063
Total			45	1.000	96	1.000

Table S6. Parameters and distributions used in the probabilistic sensitivity analysis

Parameter	Distribution
Unit Costs	Gamma
Percentage of patients using cost components	Beta
Percentage of patients thrombolysed	Beta
Utility decrement (1-utility)	Gamma
Percentage of patients 0 days LOS	Beta
First ward admitted to	Dirichlet
**Patient movements including death**	
Weibull Model – constant	Normal
Weibull Model – gamma	Normal
Weibull Model – discharge destination	Normal
Percentage that move to each location	Dirichlet

Figure S3. Distribution of patients between states from stroke onset to 90 days after stroke: Before period

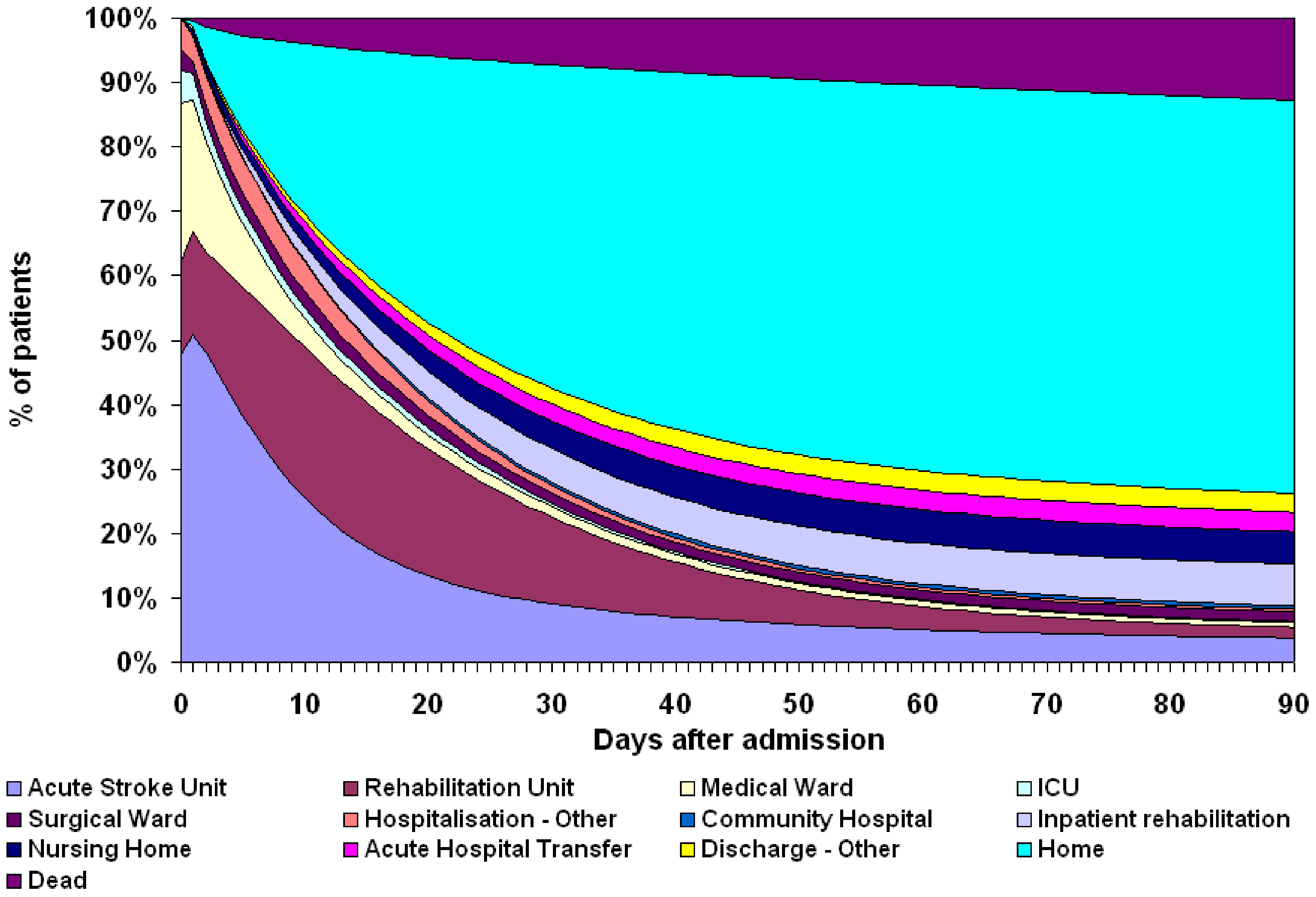

Figure S4. Distribution of patients between states from stroke onset to 90 days after stroke: After period

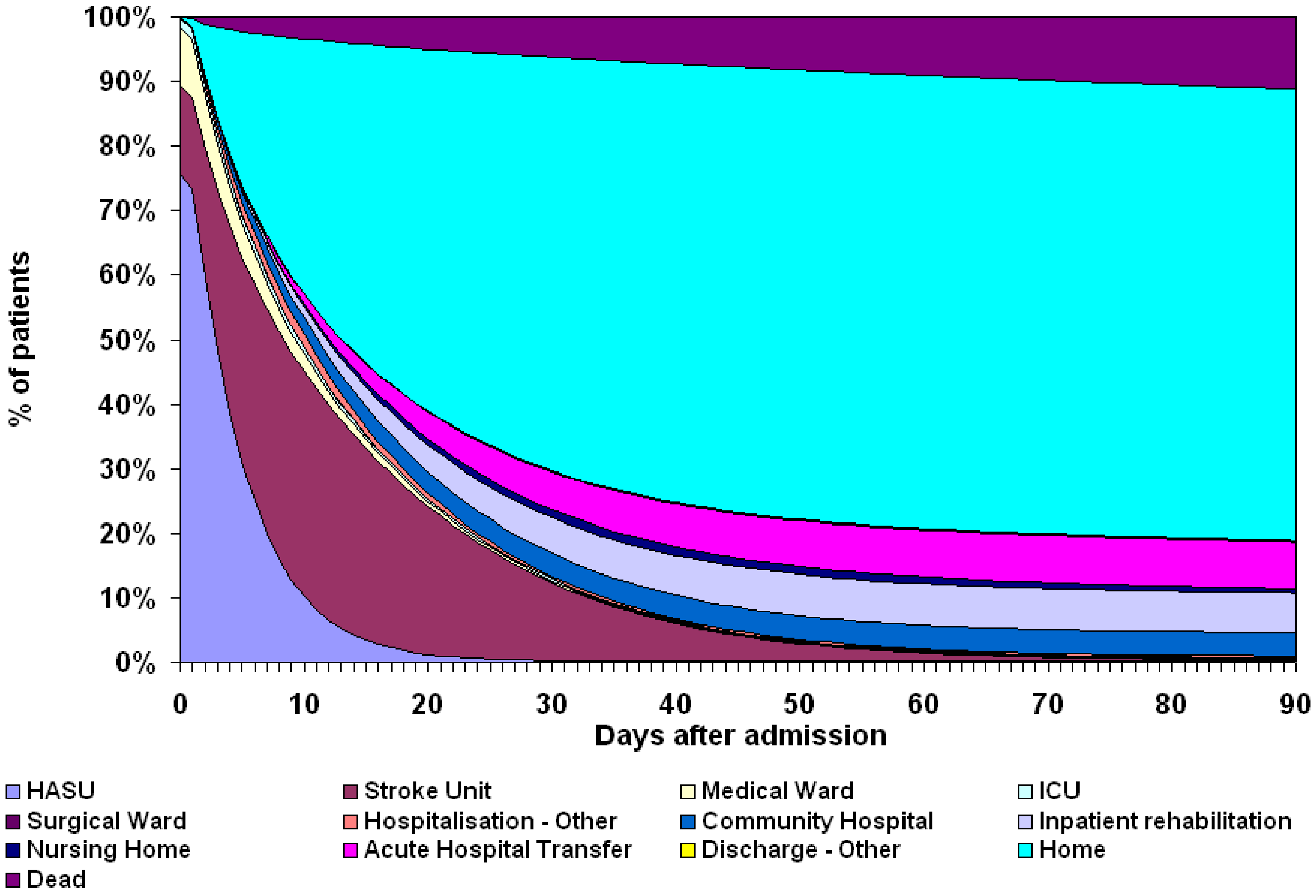

Figure S5. Distribution of patients between states from 90 days to ten years after stroke: Before period

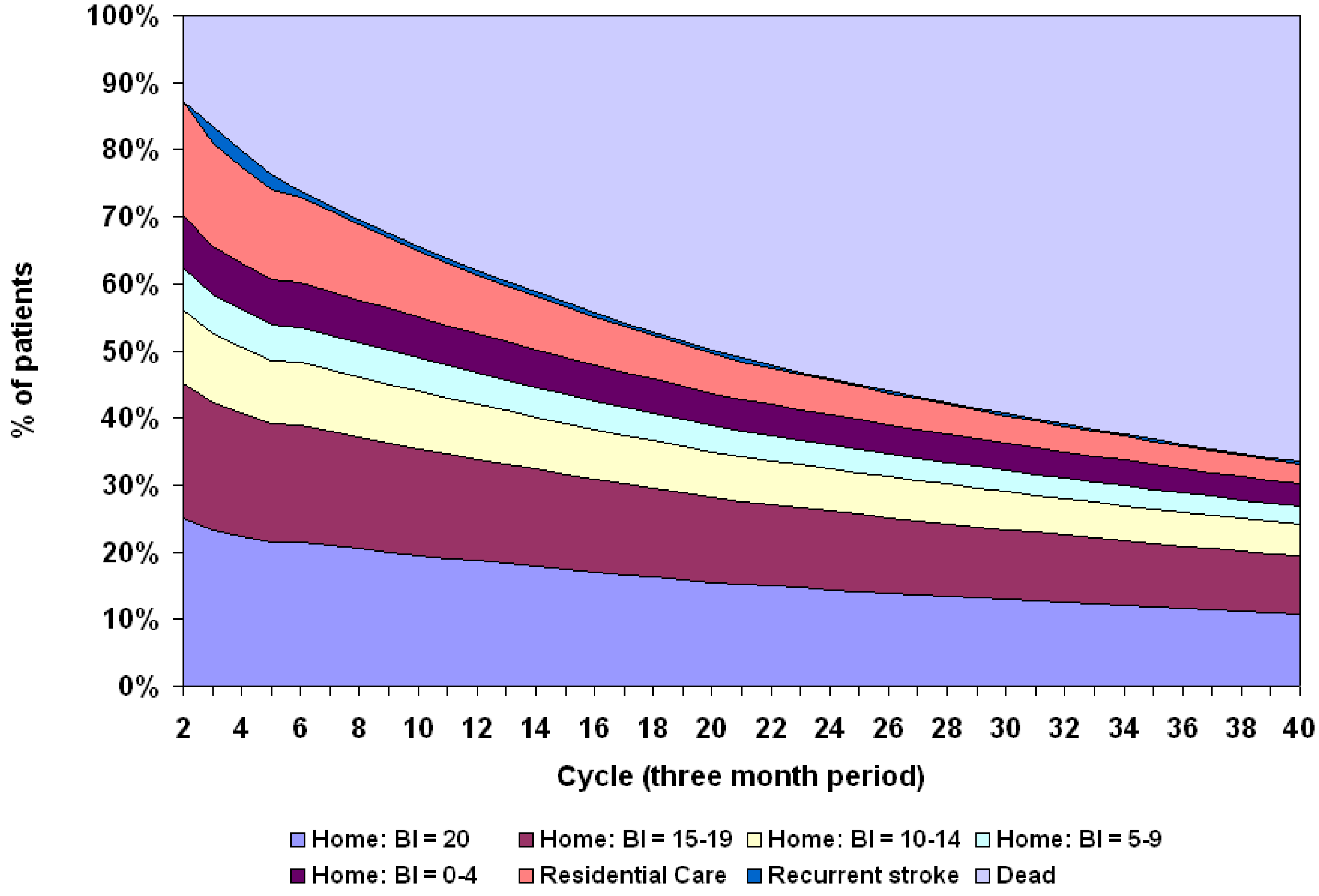

Figure S6. Distribution of patients between states from 90 days to ten years after stroke: After period

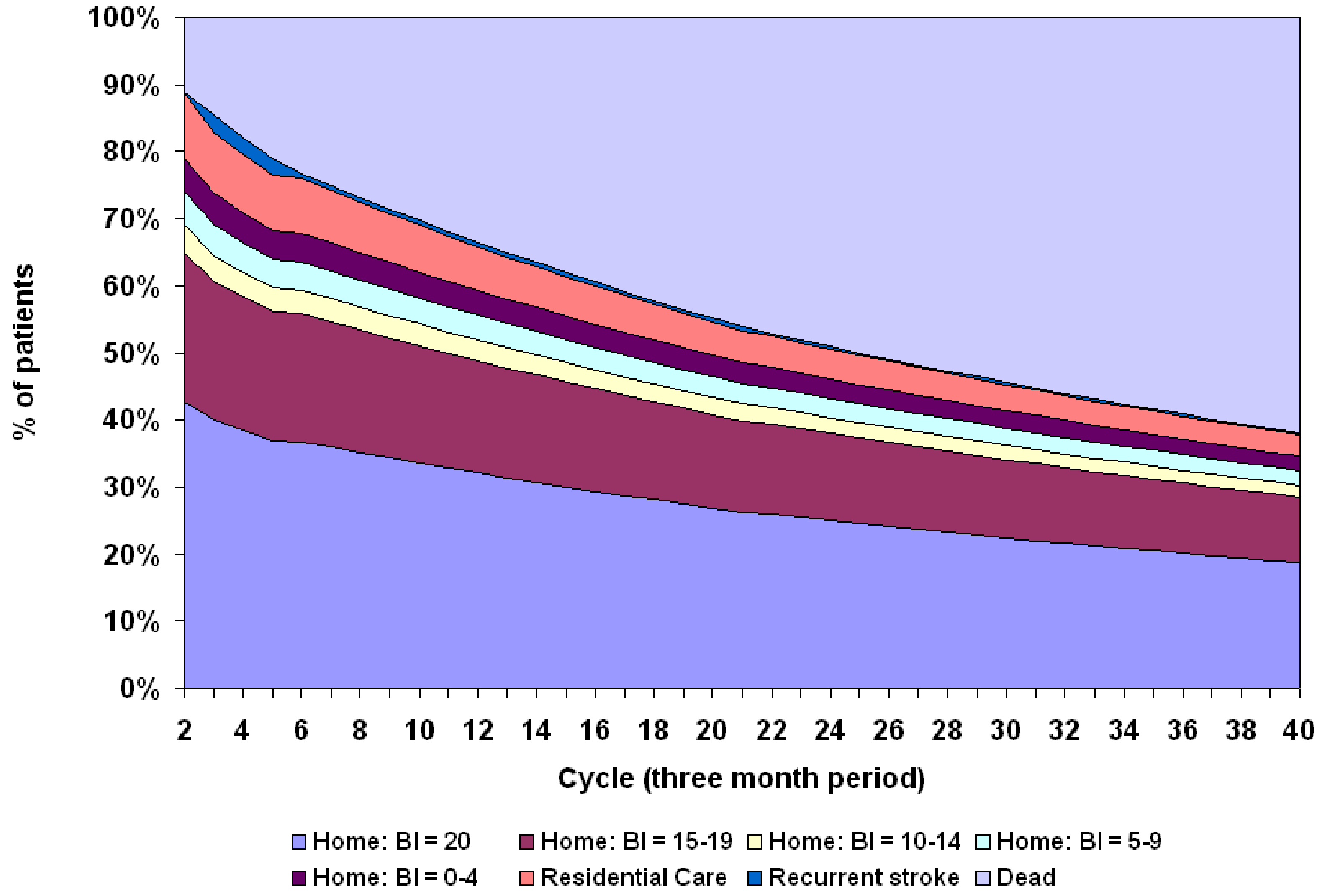

Figure S7. Monte Carlo simulations of incremental cost per QALY gained of new London stroke service using 90-day time horizon

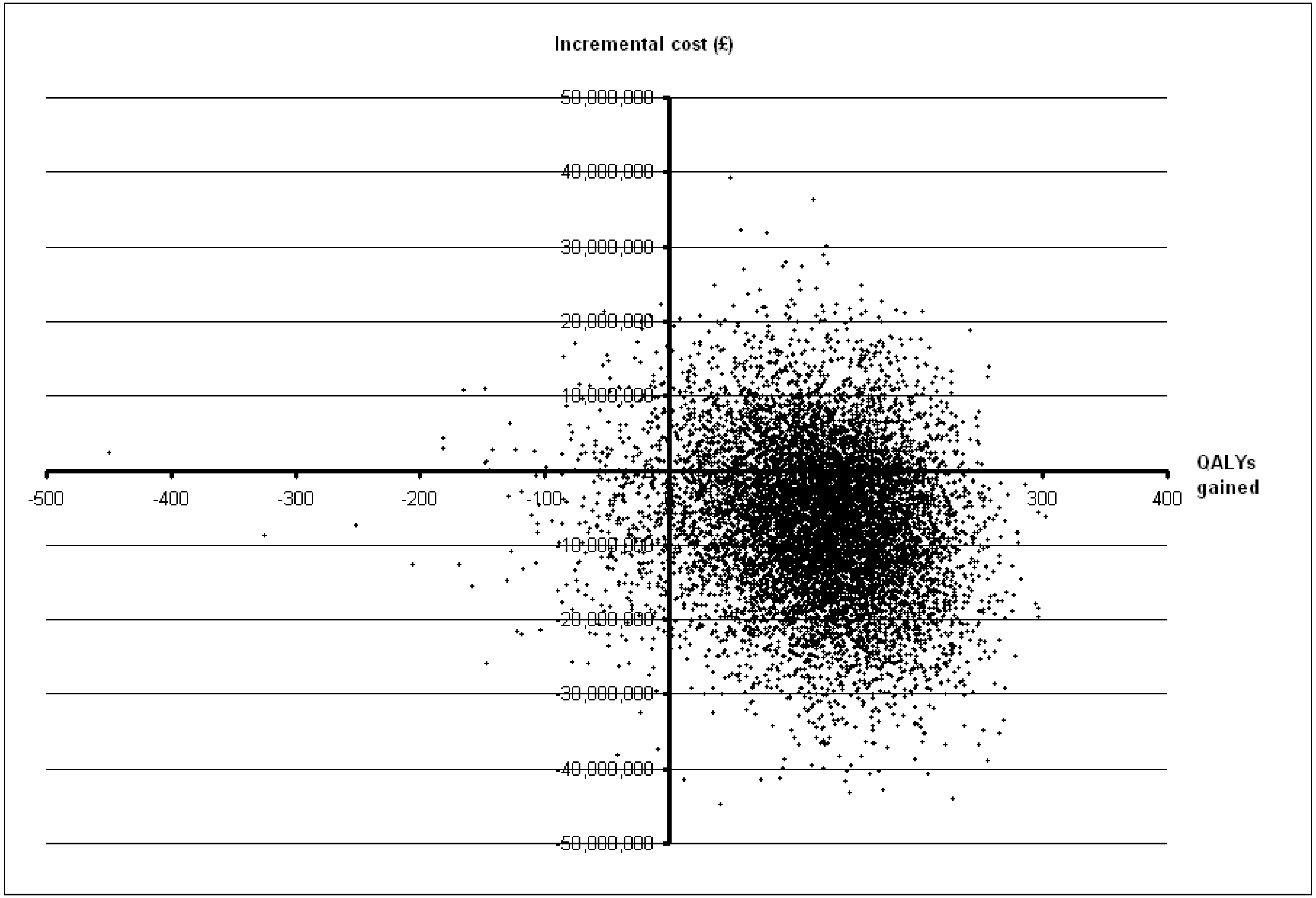

Figure S8. Monte Carlo simulations of incremental cost per QALY gained of new London stroke service using ten year time horizon

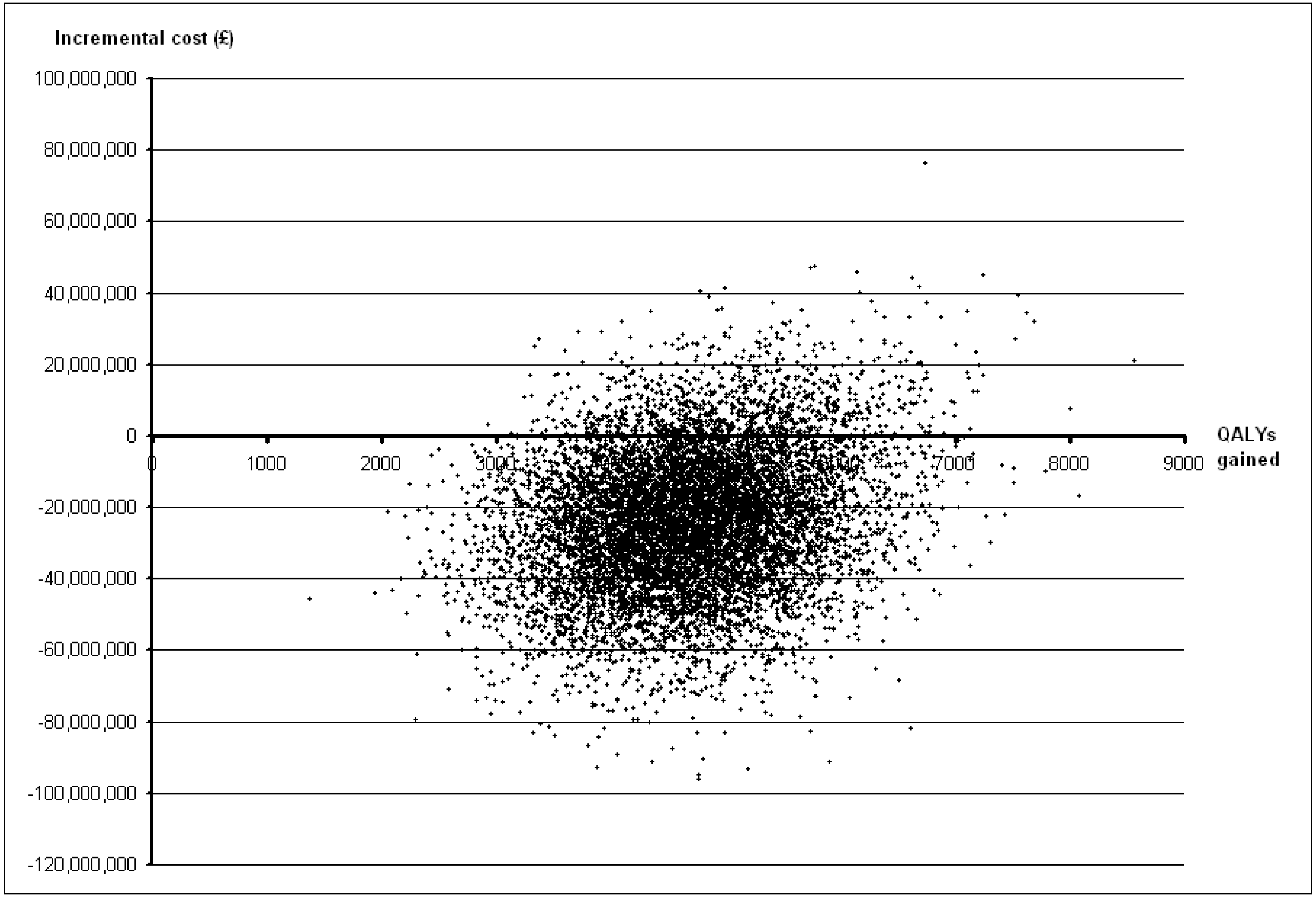

References

(1) Healthcare for London. Stroke rehabilitation guide: supporting London commissioners to commission quality services in 2010/11. Healthcare for London, editor. 2009.

(2) Care Services Improvement Partnership. Reviews of community hospital/intermediate care provision: good practice guide. 2008. Care Services Improvement Partnership.

(3) Briggs A, Claxton K, Schulper M. *Decision Modelling for Health Economic Evaluation*. Oxford: Oxford University Press, 2006.

(4) Kaambwa B, Billingham L, Bryan S. Mapping utility scores from the Barthel index. *Eur J Health Econ* 2011.

(5) Moves into residential care amongst older people in Britian. British Household Panel Survey Research Conference; 01 Jul 5; 2001.

(6) Mohan KM, Wolfe CD, Rudd AG, Heuschmann PU, Kolominsky-Rabas PL, Grieve AP. Risk and cumulative risk of stroke recurrence: a systematic review and meta-analysis. *Stroke* 2011;42:1489-1494.

(7) Wolfe CD, Crichton SL, Heuschmann PU et al. Estimates of outcomes up to ten years after stroke: analysis from the prospective South London Stroke Register. *PLoS Med* 2011;8:e1001033.

(8) Bebbington A, Darton R, Bartholomew R, Netten A. Survey of Admissions to residential and nursing home care: final report of the 42 month follow-up. 2000. University of Kent. PSSRU Discussion Paper 1675.

(9) Jones L, Griffin S, Palmer S et al. Clinical effectiveness and cost-effectiveness of clopidogrel and modified-release dipyridamole in the secondary prevention of occlusive vascular events: a systematic review and economic evaluation. *Health Technol Assess* 2004;8:iii-196.

(10) Youman P, Wilson K, Harraf F, Kalra L. The economic burden of stroke in the United Kingdom. *Pharmacoeconomics* 2003;21 Suppl 1:43-50.

(11) National Audit Office. Progress in improving stroke care: report on the findings from our modelling of stroke provision. 2010. National Audit Office.

(12) Curtis L. Unit Costs of Health and Social Care 2008. 20008. University of Kent, Personal Social Services Research Unit.

(13) DH PbR Finance and Costing Team. National Schedule of Reference Costs 2008-09. 28-1-2010. Department of Health.

(14) Kalra L, Evans A, Perez I, Knapp M, Swift C, Donaldson N. A randomised controlled comparison of alternative strategies in stroke care. *Health Technol Assess* 2005;9:iii-79.

(15) Ridley S, Morris S. Cost effectiveness of adult intensive care in the UK. *Anaesthesia* 2007;62:547-554.

(16) Mowatt G, Cummins E, Waugh N et al. Systematic review of the clinical effectiveness and cost-effectiveness of 64-slice or higher computed tomography angiography as an alternative to invasive coronary angiography in the investigation of coronary artery disease. *Health Technol Assess* 2008;12:iii-143.

(17) Curtis L. Unit Costs of Health and Social Care 2010. 2010. University of Kent, Personal Social Services Research Unit.

(18) Joint Formulary Committee. *British National Formulary*. 60 ed. London: British Medical Association and Royal Pharmaceutical Society, 2010.
